# A one-way ticket: Wheat roots do not functionally refill xylem emboli following rehydration

**DOI:** 10.1093/plphys/kiae407

**Published:** 2024-09-19

**Authors:** Beatrice L Harrison Day, Kate M Johnson, Vanessa Tonet, Ibrahim Bourbia, Chris J Blackman, Timothy J Brodribb

**Affiliations:** School of Natural Sciences, University of Tasmania, Hobart, TAS 7001, Australia; Plant Ecology Research Laboratory, École Polytechnique Fédérale de Lausanne, 1015 Lausanne, Switzerland; Swiss Federal Institute for Forest, Snow and Landscape Research, 8930 Birmensdorf, Switzerland; School of Natural Sciences, University of Tasmania, Hobart, TAS 7001, Australia; School of the Environment, Yale University, New Haven, CT 06520, USA; School of Natural Sciences, University of Tasmania, Hobart, TAS 7001, Australia; School of Natural Sciences, University of Tasmania, Hobart, TAS 7001, Australia; School of Natural Sciences, University of Tasmania, Hobart, TAS 7001, Australia

## Abstract

Understanding xylem embolism spread in roots is essential for predicting the loss of function across root systems during drought. However, the lasting relevance of root embolism to plant recovery depends on whether roots can refill xylem emboli and resume function after rehydration. Using MicroCT and optical and dye staining methods, we investigated embolism repair in rehydrated intact roots of wheat (*Triticum aestivum* L. ‘Krichauff’) exposed to a severe water deficit of −3.5 MPa, known to cause approximately 30% total root network embolism in this species. Air emboli in the xylem vessels of intact roots remained clearly observable using MicroCT after overnight rehydration. This result was verified by xylem staining of the root system and optical quantification of emboli, both of which indicated a lack of functional root xylem recovery 60 h following soil re-saturation. The absence of root xylem refilling in wheat has substantial implications for how we understand plant recovery after drought. Our findings suggest that xylem embolism causes irreversible damage to the soil–root hydraulic connection in affected parts of the root network.

## Teaser

Rehydrated roots of intact wheat (*Triticum aestivum*) plants show no evidence of functional embolism repair when using noninvasive imaging techniques and active xylem staining.

## Introduction

Despite detailed and highly informative research into plant mortality over the past decade (Choat et al. 2018; Brodribb et al. 2020; Mcdowell et al. 2022), plant roots—the all-important interface between plants and soil water resources—remain a mysterious component of plant behavior during water stress. Characterizing the dysfunction of root networks during drought has key relevance for understanding when and how plants become hydraulically isolated from the soil, with immediate flow-on effects to the water status of the whole plant and direct consequences for productivity in agricultural and natural systems (Gleason et al. 2017; Rodriguez-Dominguez et al. 2018; Peters et al. 2020). Damage to plant vascular systems during drought is largely caused by xylem embolism, whereby microscopic bubbles nucleate across xylem pit membranes under tension, causing the embolism of xylem vessels and blockage of water transport (Tyree and Sperry 1989; Brodersen et al. 2019). Acute damage to the vascular system by embolism results in downstream tissue death by desiccation (Cochard et al. 2013; Choat et al. 2018; Brodribb et al. 2021; Johnson et al. 2022; Mantova et al. 2023; Tonet et al. 2023). Much of this work has focused on stems and leaves (Cochard et al. 2015; Brodribb et al. 2016), although this theoretical framework may also be applied to underground tissues by examining vulnerability to xylem embolism in roots (Rodriguez-Dominguez et al. 2018; Harrison Day and Brodribb 2023).

Detection of embolism in roots has been made accessible through adaptations to the optical vulnerability method and X-ray computed tomography (MicroCT), building on techniques developed for observing xylem vulnerability in the above-ground tissues of plants (Cochard et al. 2015; Brodersen and Roddy 2016; Brodribb et al. 2016; Rodriguez-Dominguez et al. 2018; Lübbe et al. 2022; Harrison Day and Brodribb 2023). Due to the challenges of capturing underground traits, and difficulties with using more classical hydraulic techniques in root segments (Cochard et al. 2013; Martin-StPaul et al. 2014; Torres-Ruiz et al. 2017), root hydraulic vulnerability remains understudied. While characterizing root vulnerability (Rodriguez-Dominguez et al. 2018) and the progression of embolism through root networks (Skelton et al. 2017; Harrison Day and Brodribb 2023) provides a starting point for investigating root damage in drought, an essential functional question remains: can embolized root xylem refill when rewatered? If the damaged roots can simply repair embolized xylem after rewatering and regain function (McCully et al. 1998), embolism may have limited long-term implications to plant function after rain. However, if root xylem conduits fail to refill, then embolism has permanent consequences for the survival and recovery of plants exposed to drought.

The idea that xylem refilling and embolism repair occurs on a daily basis is a theory that has attracted considerable debate, with current consensus suggesting that, in most plants, refilling does not occur in the above-ground tissues of intact plants under field conditions (Sperry 2013; Cochard et al. 2015; Charrier et al. 2016; Lamarque et al. 2018; Choat et al. 2019). Xylem refilling has been widely debated across taxa, particularly in monocot species, with many hypothesizing that positive pressure serves to refill embolized xylem (Ryu et al. 2016; Gleason et al. 2017). However, embolism repair does not occur in leaves of wheat (*Triticum aestivum*), a monocot noted to produce guttation in leaves (and thus able to produce the positive pressure hypothesized to refill vessels) (Johnson et al. 2018). The capacity for embolism repair in roots is particularly controversial. Literature on maize suggests that root xylem routinely embolize and refill during normal daytime transpiration (Tyree et al. 1986; McCully et al. 1998; Buchard et al. 1999; McCully 1999; Facette et al. 2001; Kaufmann et al. 2009). These results appear at odds with observations in above-ground tissues (Cochard et al. 2015), although roots show marked physiological differences to leaves and stems such as their direct contact with liquid water, which create plausible conditions for embolism repair. Historically, a confounding factor in our understanding of xylem refilling has been the use of invasive methods such as cryo-SEM: a technique that has attracted debate due to the potential introduction of artifacts from freezing cut segments (Umebayashi et al. 2016; Yazaki et al. 2019). With this considered, the assumption that roots routinely refill drought-embolized xylem requires re-evaluation.

Recently available noninvasive techniques such as MicroCT and the optical method enable intact roots to be scanned in situ, allowing dehydration and rehydration to be observed in undamaged plants while minimizing methodological artefacts (Cochard et al. 2015; Rodriguez-Dominguez et al. 2018). MicroCT provides a snapshot of the physical state of the xylem during dehydration and rehydration. However, it is unable to quantify hydraulic function within those roots. Thus, additional experiments are necessary to examine root network dysfunction during drought and whether hydraulic function is recoverable following rehydration. In this study, in conjunction with MicroCT and optical methods, we use active xylem staining (Cochard and Tyree 1990; Hammond et al. 2019) to visualize the degree of root network damage over a drying and rehydration cycle to understand whether embolism results in permanent or transient dysfunction.

Wheat is an ideal candidate species for observing potential xylem refilling given its capacity to generate positive pressure within the xylem (Goatley and Lewis 1966; Johnson et al. 2018), and its well described root network and apical vulnerability to embolism (Harrison Day and Brodribb 2023; Harrison Day et al. 2023). Wheat root networks show a broad spread in vulnerabilities of the component roots. Peripheral parts of the root network contribute significantly to the water uptake of the plant, but are highly vulnerable to embolism, while the large roots show greater resistance to embolism and have a greater role in bulk water transport within the root network (Harrison Day et al. 2023). The refilling of early embolized roots could conceivably salvage the hydraulic function of the root network. Using bread wheat (*T. aestivum* L. ‘Krichauff’), an economically significant monocot species, we make a detailed in situ multimethod investigation combining MicroCT, optical methods, and safranin xylem staining, into the capacity of roots to refill xylem embolism and regain hydraulic function upon rehydration following drought. Ultimately, we ask the key question of whether xylem embolism within the roots of wheat is irreparable.

## Results

### MicroCT rehydration

**Figure 1.**
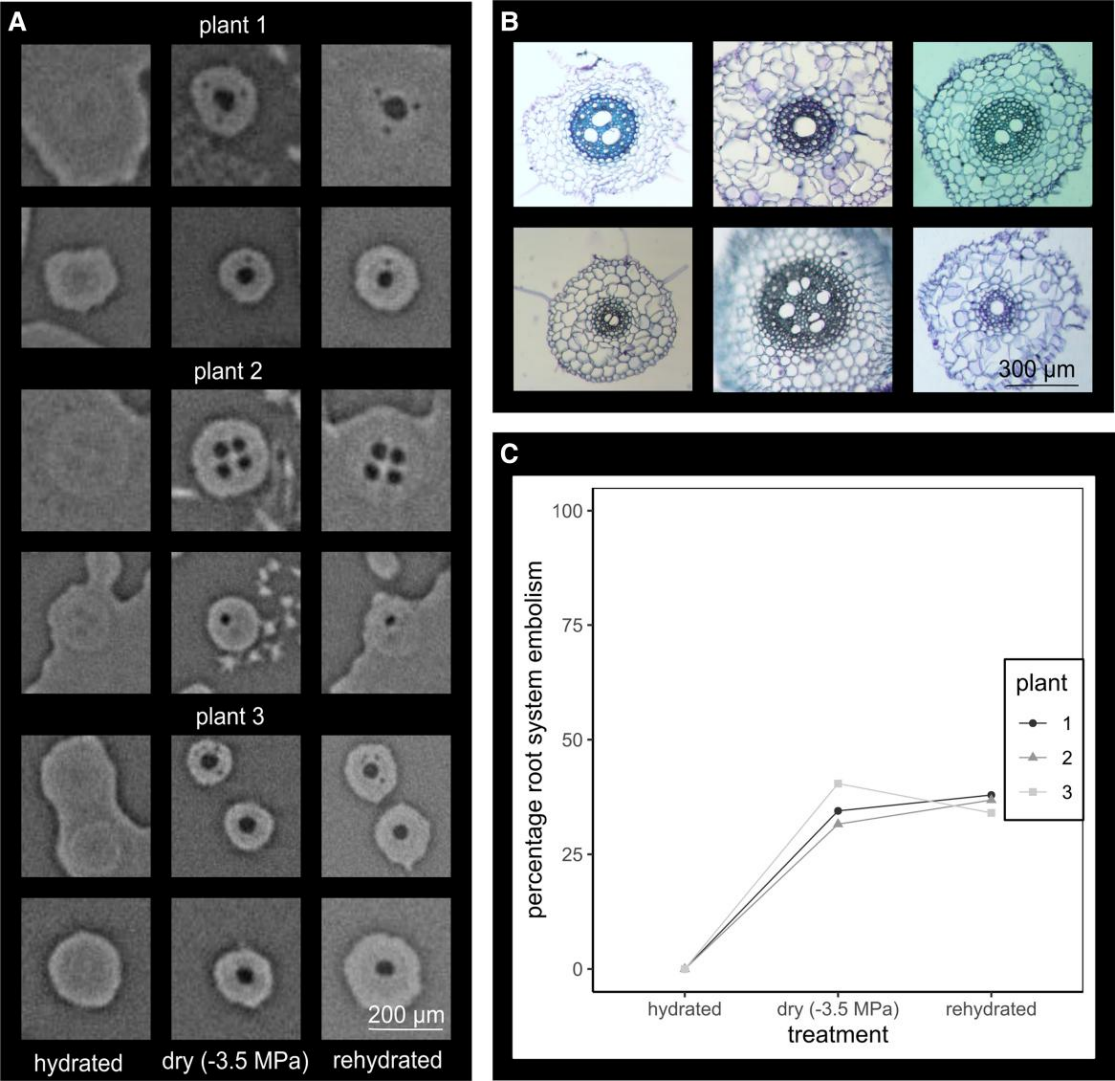
Rehydration of wheat roots. **A)** Example MicroCT scans of roots of three intact replicate wheat plants (water filled vessels gray, embolized vessels black). Scans were made when roots were fully hydrated (0.0 MPa), at −3.5 MPa where root systems were expected to show partial embolism, and then rescanned following overnight saturation (0.0 MPa). Liquid water can be seen as the lighter gray medium surrounding the hydrated and rehydrated scans. **B)** Example xylem anatomy of large roots using wet mount light microscopy, stained with toluidine blue. **C)** The proportion of embolized roots per plant across the water potential treatments of hydrated (0.0 MPa), dry (−3.5 MPa), and rehydrated (0.0 MPa).

Embolized metaxylem in intact roots of wheat could be easily differentiated from water-filled vessels using MicroCT following an imposed water stress of −3.5 MPa. After 12+ hours overnight rehydration, embolized central metaxylem remained clearly observed in the corresponding root scans, even when surrounded by liquid water (Fig. 1). Using MicroCT, embolisms within the central metaxylem could be resolved in a total of 37 roots out of 95 large roots across the three intact plants at −3.5 MPa, with smaller roots not resolvable due to pixel size. Emboli remained clearly visible (indicating no evidence of xylem embolism refilling) in 34 out of the 37 embolized roots (Fig. 1), with two plants showing an additional embolized root in the subsequent scans. Emboli could not be identified in three roots in the subsequent rehydrated scan of one plant. These MicroCT data should be considered with a degree of caution in case repeat X-ray exposure may have impeded cellular processes associated with active water uptake in living root cortical cell layers.

### Dye rehydration experiments

**Figure 2.**
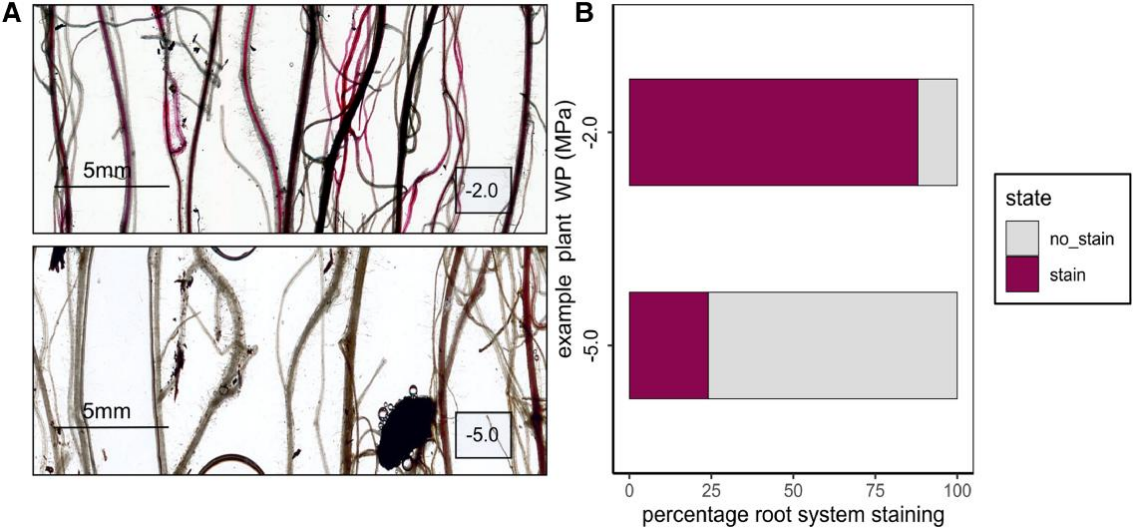
Active xylem staining of wheat roots in two example plants using safranin O, following 12+ hours overnight staining via cut stems, to demonstrate xylem function across the root network. **A)** Example subset scans of stained (functional) and unstained (nonfunctional) roots of plants stained at −2.0 and −5.0 MPa dehydration treatments. **B)** Percentages of staining across the root network of the two plants.

Safranin xylem staining via cut stem rehydration over 12+ hours was highly successful in demonstrating the proportion of dysfunction across the intact root networks of wheat plants exposed to drying (Fig. 2), with roots with functional xylem (magenta) easily differentiated from nonfunctional (white) using transmitted light. In bare rooted plants (three plants per treatment, 50 roots per plant) the percentage of stained (functional) roots declined from 94 ± 3 SD % in roots exposed to −0.9 MPa, to 86 ± 1% at −2.0 MPa, 73 ± 5% at −3.5  MPa and to 16% stained roots at −5.0 MPa.

**Figure 3.**
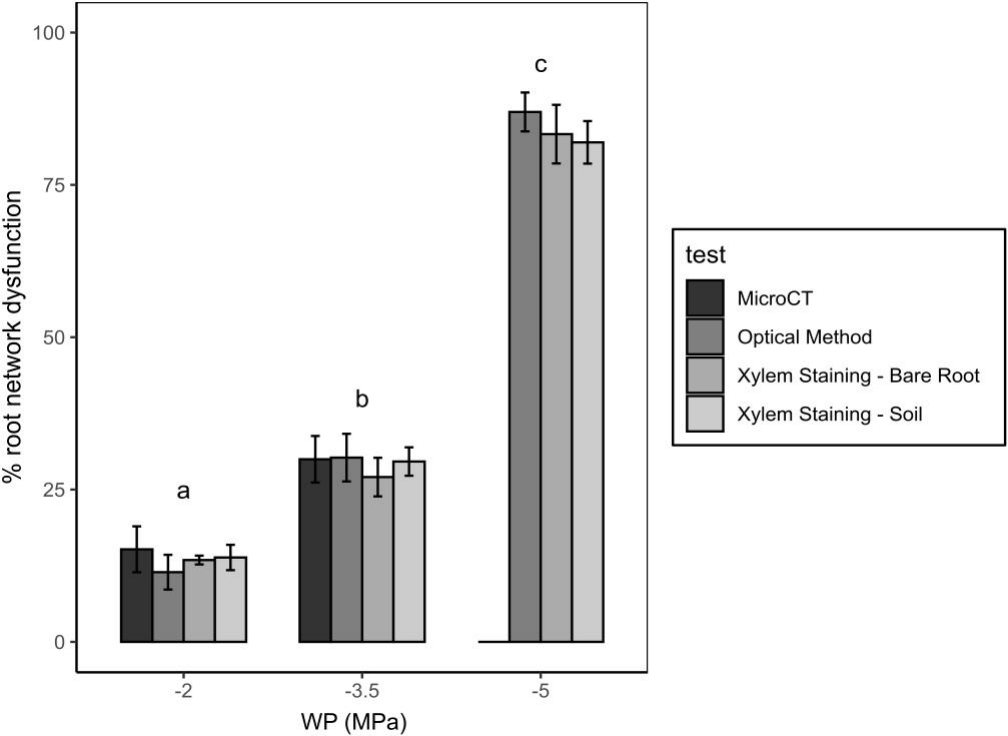
Percentage estimates ± Se of root network dysfunction across the roots of wheat using MicroCT (% root network embolism), the optical method (OV) (% root network embolism), and active xylem staining (% unstained roots following 12+ hours overnight intact root network staining via cut stems). Letters indicate significant differences (*P* < 0.05) across the water potential treatments of −2.0, −3.5, and −5.0 MPa (calculated using two-way ANOVA of unequal sample size). No significant differences were observed in the estimates of network function across the methods within treatments. MicroCT and OV root values taken from Harrison Day et al. (2023) on plants of the same genotype and developmental age. WP (MPa) = water potential thresholds used across experiments.

The estimated percentage dysfunction of the root network (as indicated by the percentage of unstained roots) over progressively dry water potential treatments was highly consistent across methods, with increasing water stress having a significant effect on the decreasing percentage of root network function (*P* < 0.001). Xylem staining values showed no significant differences (*P* > 0.5) from the known percentage root network embolism predicted by the optical method and MicroCT (Harrison Day et al. 2023) of the same genotype (Fig. 3). All techniques similarly estimated ∼15% loss of root network function through embolism at −2.0 MPa, 30% loss of function at −3.5 MPa and 85% loss of function at −5 MPa, respectively. There was no significant difference between the stain results in soil or in bare rooted plants, suggesting that root damage was minimal during soil removal.

**Figure 4.**
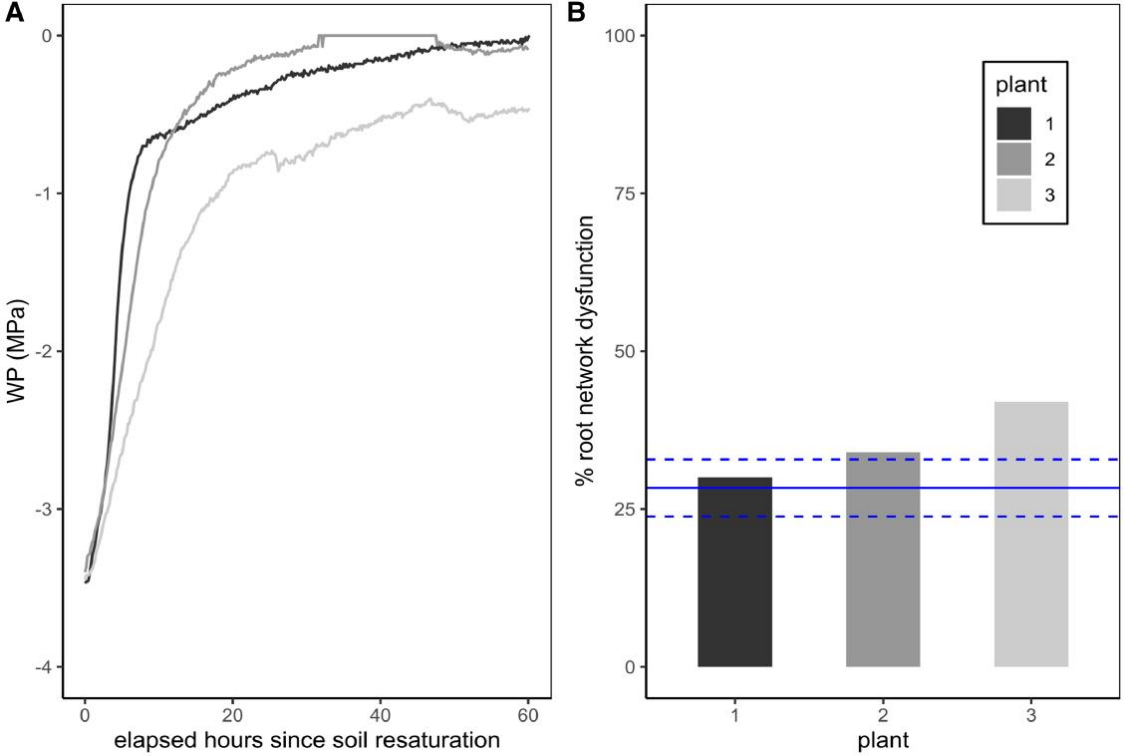
Recovery of intact roots after stress and rewatering in soil of wheat following an imposed water stress of −3.5 MPa, measured using a stem psychrometer. **A)** Recovery of water potential in three replicate wheat plants following the re-saturation of soil for 60 h. **B)** Corresponding estimates of root network dysfunction (% of roots that failed to stain) of the same plants using safranin xylem staining, showing no recovery of function in the roots beyond projections expected through xylem embolism (blue line = mean % embolism taken from xylem staining methods ± Sd as dashed blue lines). WP (MPa) = water potential.

Plants dried in soil to the threshold of −3.5 MPa and then re-saturated for 60 h in soil showed a mean 35.33 ± 6.11 SD % root network dysfunction (indicated by roots failing to stain). The mean % embolism at −3.5 MPa predicted from all xylem staining values in-soil and in bare-rooted plants was 28.33 ± 4.53 SD %, so in the case of embolism repair post-rehydration this number was expected to decline, thus regaining function. On the contrary, all re-saturated plants showed dysfunction equal to or greater than the % embolism expected, revealing an additional slight loss of root function after rehydration rather than xylem embolism repair in these plants (Fig. 4).

### Optical method rehydration experiments

**Figure 5.**
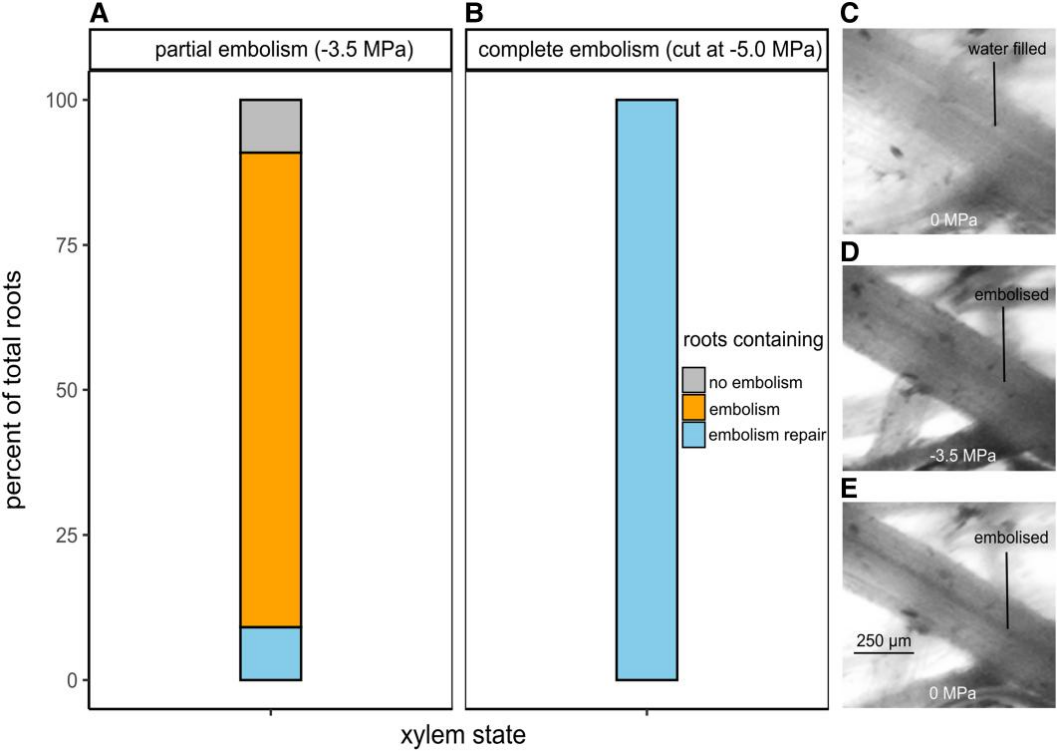
Optical assessment of root xylem rehydration. Xylem embolism and rehydration of wheat roots to investigate embolism repair in plants dried to −3.5 MPa **(A)** and −5.0 MPa (cut to ensure complete embolism) **(B)** using optical methods. **A, B)** Percentages of total root outcomes per −3.5 and −5.0 MPa treatments (three plants per water potential treatment, 6 to 8 root segments per plant, 22 root segments total per water potential treatment). **C** to **E)** Dehydration and re-saturation of an example root segment dried to −3.5 MPa, showing partial root embolism, with embolized xylem remaining in situ following re-saturation. Scale applied to all images.

Using the optical method, xylem embolism refilling was not observed in the embolized roots when neighboring regions of root were saturated with water following exposure to water potentials of −2.0, −3.5, and −5.0 MPa (Supplementary Fig. S3). Although abundant water was provided adjacent to the cameras (<5 cm from the site of rewatering) and to the rest of the root mass, embolized roots were unable to regain water transport to refill root xylem contained within the cameras closely adjacent to the rewatered root segments (Supplementary Fig. S4). Xylem embolism refilling was not observed in 81.82% of the roots of plants dehydrated to −3.5 MPa when rewatered directly onto the glass slide containing roots of interest, with the exception of two root segments out of a total 22 observed (9.09%), and 2 root segments showed no degree of embolism (9.09%) (Fig. 5). Xylem refilling could be observed locally in all fully embolized roots cut at −5.0 MPa when rewatered directly onto the glass slide. Thus, across all experiments, embolism refilling was only consistently observed when roots were fully embolized, cut and in direct contact with water (Fig. 5) (Supplementary Video 1).

## Discussion

Across our varied experiments we found that functional xylem embolism repair did not occur following rehydration of water-stressed roots of wheat. Xylem embolism remained visible in MicroCT scans of roots saturated overnight following an imposed stress known to cause 30% root network embolism (although the scanning of living material may have impacted root function). This result was confirmed using a dye perfusion method, showing that loss of function in roots subjected to water stress in soil showed no evidence of embolism repair in the 60 h following rewatering. On this basis we suggest that the embolism of wheat roots results in permanent dysfunction of the affected roots.

Wheat is an ideal candidate for observing xylem embolism refilling given its ability to generate considerable root pressure capable of producing guttation in leaves, a trait hypothesized to allow the repair of embolisms in xylem (Goatley and Lewis 1966; Singh 2013; Gleason et al. 2017; Johnson et al. 2018; Schenk et al. 2021). Although we did not find evidence for functional embolism repair in plants returned to hydrated water potentials, these plants were not observed to produce guttation. Neither our bare-rooted or soil rehydrated experiments produced guttation, either due to the timescale of rehydration, or due to the proportion of embolism within the whole plant (although truly quantifying positive pressure is a destructive process; Clearwater et al. 2007). This reinforces that embolism repair through positive pressure is not a rapid and immediate process in this species but highlights that until plants can be forced to produce guttation and then be assayed for root refilling, this condition cannot be discounted.

Root refilling either during or after exposure to water stress is a persistent theory proposed to explain plant survival following tissue embolism (McCully et al. 1998; Buchard et al. 1999; McCully 1999; Nardini et al. 2011). Wheat is a species that shows dramatic vulnerability segmentation across the many component roots of the root system (Harrison Day and Brodribb 2023; Harrison Day et al. 2023). Peripheral fine roots grow extremely rapidly to take up water and nutrients but are highly vulnerable to embolism, showing carbon allocation toward accelerated growth rather than xylem resistance. The large roots, and regions closest to the root collar, show significant resistance to embolism and are the longest lived part of the root network, revealing carbohydrate investment in construction of embolism resistant xylem (Harrison Day and Brodribb 2023; Harrison Day et al. 2023). The loss of the fine roots during drought imposes a significant resource cost to the plant to regrow and regain network function. Thus it seems that the repair of embolized roots would be an energetically favorable mechanism to salvage the complete hydraulic function of the root network compared to root regrowth. Our evidence, however, shows that embolism repair in roots is not routinely occurring, with plants instead relying on the proportion of highly resistant roots to maintain a last line of hydraulic defense until the death of all apical parts of the plant (Rodriguez-Dominguez et al. 2018; Harrison Day and Brodribb 2023; Harrison Day et al. 2023). Work on other angiosperms using noninvasive techniques also shows that embolism resistance, at least in some parts of the root system, can match or exceed that of stems and leaves (Skelton et al. 2017; Rodriguez-Dominguez et al. 2018; Peters et al. 2020; Lübbe et al. 2022), suggesting that embolism resistant roots may be relatively common. The lack of root embolism repair seen here mirrors observations from above-ground tissues that suggest xylem refilling does not occur in most species (Delzon and Cochard 2014; Charrier et al. 2016; Lamarque et al. 2018; Choat et al. 2019). Although these wheat results may not reliably be extrapolated to broader species, it is particularly significant to find a lack of refilling in a species exhibiting traits that should facilitate embolism repair, and in a species so key to global food supply.

A number of studies in maize roots suggest that root xylem routinely become embolized during hot and dry days and vessels refill again by sunset (Tyree et al. 1986; McCully et al. 1998; Buchard et al. 1999; McCully 1999; Facette et al. 2001; Kaufmann et al. 2009) with some even showing that refilling can occur on a timescale of minutes (Facette et al. 2001). These papers, and others on soybean, buckwheat and a neotropical savanna species (Buchard et al. 1999; Domec et al. 2006) credit a complex mix of root pressure and phloem unloading for refilling embolized xylem (Nardini et al. 2011), with root refilling a pervasive and long-standing idea (Tyree et al. 1986; Ewers et al. 1997; Johnson et al. 2018). In one report (Shane and Mccully 1999), maize roots were observed to reach 40% to 60% metaxylem embolism by midday at water potentials of only ∼−1.0 MPa and refilling by sunset when water potentials had relaxed to −0.4 MPa. These experiments, however, utilized cryo-scanning electron microscopy (cryo-SEM) on cut root segments, a technique that has been queried for potentially overstating the degree of embolism refilling in other plant tissues (Cochard et al. 2000; Cochard and Delzon 2013; Gleason et al. 2017).

While cryo-SEM assessments of embolism repair may be problematic, other estimates such as loss of conductivity and MRI, also in maize, provide evidence for xylem refilling in roots that appears more robust (Kaufmann et al. 2009; Gleason et al. 2017). This suggests that either maize and wheat display divergent root hydraulic vulnerability traits, despite their physiological similarities as monocot species, or that the refilling signals observed by Kaufmann et al. (2009) may reflect a unique set of circumstances. We were able to force xylem refilling in one very limited scenario using the optical method by direct rewetting of cut, fully embolized roots directly onto the field of view, and in two root segments out of 22 of the rehydration experiments from −3.5 MPa (at water potentials beyond the embolism of leaves; Harrison Day and Brodribb 2023). However, fully embolized roots saturated <5 cm adjacent to cameras in roots at −3.5 and −5.0 MPa showed no sign of embolism repair and thus no recovery of functional water transport. Importantly, if refilling were to have facilitated the functional repair of xylem in intact roots, the percentage root network function recorded in our xylem staining methods using plants in saturated soil would have theoretically exceeded the amount seen across the embolism experiments. In the 60-h soil rehydration experiments, and in MicroCT, the xylem emboli were not repaired despite the return to hydrated water potentials, indicating that embolism in the roots resulted in irreparable damage to xylem function. Three embolized roots out of the total 37 could not be located in our MicroCT scans following rehydration, however, the lack of evidence for functional recovery in the soil rehydration experiment over the longer timescale suggests that these three roots may not have been indicative of functional embolism repair. The NMR evidence provided by Kaufmann et al. (2009) showing embolism repair in some but not all maize roots was not paired with water potential, so the severity of the drought stress prior to rehydration is unknown and may be more similar to the processes observed in cut roots here, although this may still reflect differences between maize and wheat. Recently developed techniques like MicroCT and xylem staining provide the opportunity to test these ideas more broadly.

A lack of embolism repair suggests that xylem embolism in roots represents a permanent physiological barrier to the movement of water into the plant. The irreparable dysfunction of embolized roots has serious implications for the recovery and long-term viability of plants following severe drought conditions. Here, plants must either operate with impaired root hydraulic conductivity or regrow the lost roots to regain root network function at significant carbon cost (Brunner et al. 2013; Bourbia et al. 2021). For plants that persist over multiple seasons, new roots may recover conductance if the meristems remain intact and the plants retain enough carbon resources (Ruehr et al. 2019). In many crop species and annual plants that grow for a single season, the timing of lethal root embolism is thus essential to the viability of the plant (Hahn et al. 2021; Harrison Day and Brodribb 2023). Retaining a cohort of resistant roots is vital if the plants need to rehydrate following extreme drought.

Our findings that roots cannot refill embolized xylem emphasize the importance of examining underground hydraulic traits during drought. According to these results, measuring root embolism is crucial for characterizing how plants become disconnected from soil water resources under progressive drought. Here we show that embolism of individual wheat roots is a one-way ticket to the death of that root segment, a finding which has key implications for understanding this important crop species and warrants further investigation across diverse plant species.

## Materials and methods

### Plant material and preparation

Wheat (*T. aestivum* L. ‘Krichauff’) seeds were germinated in 2 L pots filled with 7:4 loose soil mix of composted fine pine bark and coarse washed river sand and a slow-release general fertilizer (Osmocote). Forty-two plants were used across all tests (three for MicroCT, 15 for the OV method, and 24 across the active staining tests), with staggered growing times. Plants were grown in glasshouse facilities at the University of Tasmania, Australia in average day/night temperatures of 20 °C/15 °C in an 18-h photoperiod and were watered daily. Plants were grown for 8+ weeks (to approximately 40 cm in height) until early reproductive maturity but before pot-binding of roots.

Across experiments, water potential treatments of −2.0, −3.5, and −5.0 MPa were chosen to represent thresholds of xylem damage in roots and leaves at significant points during the dehydration of wheat plants. The OV and MicroCT values taken from Harrison Day et al. (2023) were achieved using identical *T. aestivum* L. ‘Krichauff’ plants at the same developmental stage. At treatment 1 (−2.0 MPa), based on the data of Harrison Day et al. (2023), on average the apical parts of the plant remain fully intact but some roots (∼15% root network embolism) show some degree of partial xylem embolism—a point where post-rewatering root refilling could theoretically recover full hydraulic function of the entire plant. At treatment 2 (−3.5 MPa), on average the majority of leaf embolism is complete and around 30% of the roots within the network show some embolism, but sufficient root function is retained to sustain flowering and theoretically support resprouting of new leaves, making this a key threshold for plant reproductive recovery should root refilling occur. At treatment 3 (−5.0 MPa), water potentials are lethal to most above-ground tissues and cause 85% root network embolism. From this level of drought stress root refilling after rewatering may theoretically facilitate resprouting but is unlikely to lead to the recovery of above-ground tissues.

### Measurement of water potential

All dehydration experiments occurred under stable lab conditions (around 30 *µ*mol quanta m^−2^ s^−1^ of light, at 18 °C temperature). Water stress in roots was generated by carefully removing intact plants from pots, and from soil in specific cases, to increase the rate of drying and gain access to roots for experimental techniques. The unique rate of water potential decline during dehydration was measured for each plant using a stem psychrometer (ICT international, Armidale, NSW, Australia) placed on the peduncle of the plant (see Harrison Day and Brodribb (2023) for greater detail). Considering wheat plants placed under these conditions begin closing stomata at −0.6 MPa and completely close stomata around −1.3 MPa, with leaf turgor loss at −0.9 MPa (Corso et al. 2020) the water potential gradient between root and stem xylem beyond −0.9 MPa would be minimal (in the absence of significant xylem damage). Embolism resistant peduncles persist longer than more vulnerable leaves during dehydration (Harrison Day and Brodribb 2023): thus, we used peduncle water potential as a reliable estimate of root water potential. This method was used on all plants measured.

### Microcomputed tomography

Synchrotron-based MicroCT was used to visualize root xylem embolism during dehydration and rehydration in three intact plants at the ANSTO Australian Synchrotron in Clayton, VIC, Australia. At the University of Tasmania, plants were removed from pots and their roots were gently rinsed with water to remove most of the soil. Whole plants were then wrapped in damp paper towel and enclosed in Ziplock bags to prevent drying, and then flown to the ANSTO Imaging and Medical Beamline (IMBL) in Clayton, Australia for MicroCT imaging. Once at the Synchrotron facilities, intact roots of each plant were carefully bundled together in parallel so they could be imaged in a single scan. This beamline has relatively low resolution but a large hutch size, allowing whole plants including roots to be studied intact. The plants were gently bound to a carbon-fiber rod using paper tape, with root bundles wrapped with paper towel and bound with parafilm and clear tape. The root scan site was marked with white correction fluid for reproduceable imaging of the same site at sequential water stress treatments. Plants were attached to a rotating stage and positioned in the beamline to target the correction fluid marked region on the roots, ∼5 cm below the root collar. The IMBL was used with an X-ray energy of 40 keV, producing a reconstruction resolution of 7.5 *μ*m per pixel. While scanning, the stage rotated at 180°, with images collected every 0.1 s. A field of view of 18.7 mm in *X* and *Y* orientations and 15.6 mm in *Z* vertical orientation were scanned, with a total scan time of ∼11 min.

In each of three replicate plants, scans were made of the intact root network at three sequential levels of water status: fully hydrated, dehydrated to −3.5 MPa (treatment 2 described above), and 12 h post-rehydration. The first fully hydrated scan assessed whether embolisms were introduced during preparation. Following the initial scan, each plant was removed from the scanning stage and a stem psychrometer attached to the peduncle (see above). Plants were then dried to a target water potential of −3.5 MPa, expected to cause ∼30% root system embolism (Harrison Day et al. 2023) and rescanned at the correction fluid marked site (after removing the psychrometer). The psychrometers were then repositioned onto the peduncles and the root bundles immersed in water in a Ziplock bag with the leaves covered in damp paper towel and bagged and allowed to rehydrate for 12+ hours overnight, reaching stable hydrated water potentials close to 0 MPa. Once rehydrated, the psychrometers were removed and a further rehydrated scan was produced for each plant to assess xylem for refilling.

### MicroCT image analysis

Scans were compiled as vertical stacks of 8bit images and analyzed in ImageJ (NIH), and a common scan site was identified across all treatments by finding the correction fluid marked region. Roots of each plant with embolized xylem were identified in the dry (−3.5 MPa) treatment, with each scan per plant containing ∼35 resolvable roots. The same roots were then identified in the following rehydrated scans, and the embolized xylem (indicated by a black void) within each root were counted again to determine whether any embolized xylem had refilled with water (gray). Given the limited pixel size, embolism was scored by number of roots containing emboli (presence/absence) rather than number of embolized conduits within roots. Percent root system embolism refilling was calculated as the total number of roots containing emboli in a scan across a single plane as a percentage of the number of resolvable roots.

### Root xylem anatomy

A selection of roots of varying diameters and positions within the root network were sectioned and imaged using light microscopy to contextualize the xylem anatomical variation visualized in MicroCT scans. Transverse sections 20 to 30 *µ*m thick were produced using a sliding microtome (Leica Microsystems, North Ryde, Australia) and BFS-3MP freezing stage (Physitemp Instruments, Clifton, USA), encased in dilute glucose solution.

### Active xylem staining

Active xylem staining methods (Cochard and Tyree 1990; Hammond et al. 2019) were devised firstly to visualize the degree of function within the root system xylem over increasingly severe water deficit, and principally as a method to determine whether roots damaged by water stress were able to recover function following the rewetting of soil over 60 h. Using 24 new plants of the same developmental stage, three experiments were designed to examine the success of root xylem staining in bare rooted plants, in soil, and the capacity for embolism repair following re-saturation in soil. The principle of the active xylem staining technique was to use the water potential gradient between submerged stems, cut just above the root collar, and the intact root network under targeted water stress. This gradient should draw water into the root along the functional xylem pathway, with staining indicative of root function. Xylem staining was conducted using a solution of 0.1% w/v safranin (Safranin O, Sigma–Aldrich, NSW, Australia) in 95% v/v filtered water and 5% v/v ethanol.

It was important to confirm that results from soil-free scanned roots were not an artifact of soil removal. For the first experiment, 12 well-watered plants were gently removed from pots, carefully shaking the soil from the roots, and allowed to dry flat under controlled lab conditions. An initial control (un-embolized) group of three bare rooted plants was dried under lab conditions to −0.9 MPa: a theoretical threshold where stomatal closure would ensure that the water potential in the roots was homogenous with the rest of the plant (Corso et al. 2020), but before significant root network embolism (Harrison Day et al. 2023). Once the plants had reached the target water potential, the stems of the plants were submerged (avoiding water/stain contact with roots) in a petri dish of safranin solution and cut near the root collar under solution with a sharp razor blade. The stems were then recut 2 to 3 cm from the root collar under the solution and maintained immersed in the stain. The intact roots were left to rehydrate via the cut stems for 12+ hours overnight. Once the control at −0.9 MPa was shown to successfully produce root network staining above 90%, the procedure was repeated on bare rooted plants (*n* = 3 per treatment) dried to each of the water potential treatments (−2.0, −3.5, −5.0 MPa).

For the second experiment, the procedure was repeated on nine plants allowed to dry in pots with soil intact (*n* = 3 plants for each of the above water potential treatments) to exclude the possibility of damage through soil removal. Potted plants were placed in a warm glasshouse (25 °C/15 °C day/night with a 12 h photoperiod) to accelerate drying in soil. Once within 0.5 MPa of the target water potential predawn, the plants were moved to stable lab conditions (see above) to allow the psychrometers to stabilize, and allowed to dry until the plants reached the target water potential thresholds. Intact plants with soil volume were transferred to shallow vessels to allow the stems to be laid flat for immersion during staining. Dye rehydration was completed as above but without removing roots from the soil.

For the experimental test of embolism repair, three plants were dried in pots under warm glasshouse conditions until close to the target water potential before being moved into stable lab conditions. Once at the target of −3.5 MPa, plants were watered to saturation and allowed to rehydrate for 60 h. This time period was longer than the xylem refilling times documented by earlier papers (Tyree et al. 1986; McCully et al. 1998; Buchard et al. 1999; McCully 1999; Facette et al. 2001; Kaufmann et al. 2009) to allow for the full capture of potential refilling, and longer than the demonstrated bare-root rehydration time (Supplementary Fig. S1), but shorter than the time needed to regrow new roots (Volaire and Norton 2006; Stewart and Frank 2008; Zwicke et al. 2015). Once fully saturated, roots were washed of soil and all plants were allowed to dry back to −0.9 MPa to facilitate the movement of dye into the roots and were stained as above.

### Xylem staining image analysis

To determine the percentage of the root system with active xylem as shown by the dye uptake, a region of the root bundle was selected 5 cm below the root collar (to mirror the MicroCT methods) for each plant. A 3 cm longitudinal section was cut from the root bundle encompassing all roots and was transferred to a petri dish of water for separation and cleaning. The clean root segments were arranged on a glass plate with a cover glass plate and were scanned in color using an Epson Perfection V800 Photo Scanner (Epson, Nagano, Japan) at 2,400 dpi. Using ImageJ, roots were classed as either stained (magenta) or not stained (white), falling into two clear categories, and were measured by diameter. Given that embolism can only be resolved in the largest 50 roots using the optical method and largest ∼35 using MicroCT (Harrison Day et al. 2023), a percentage of root network staining was calculated using the largest 50 roots per plant. This root cohort was chosen to match the root sizes examined by Harrison Day et al. (2023). The only root class excluded from tests were the smallest and finest roots and root hairs, firstly to avoid roots with nonfunctional xylem that were still growing, and secondly to avoid confounding xylem occlusion (from safranin crystals or particulates from the cut stem) with embolism in the finest roots with very small xylem (Harrison Day et al. 2023).

### Optical methods

Two experiments (direct rehydration and indirect rehydration) were conducted using optical methods (Brodribb et al. 2016) to determine the capacity of roots to refill embolized xylem via localized and adjacent re-saturation (Supplementary Fig. S2). Fifteen plants were used across all experiments, with five separate treatments designed to examine direct and indirect rehydration across different water potential thresholds, using three plants per treatment (Supplementary Fig. S2, A to E) (treatments listed below). All plants were carefully removed from pots and the root systems gently rinsed to allow multiple cameras to be fitted directly to exposed roots. Whole plants with exposed roots were laid flat and allowed to dehydrate under stable lab conditions over the course of 3 d. Two cameras (cavicam.co) were placed in series along the length of three to four intact undamaged seminal roots (∼30 cm total length, ∼10 cm between each camera) of each plant, with regions of root within the cameras placed between glass slides to prevent distortion. Roots were illuminated with white LED light to capture root xylem embolism (Brodribb et al. 2016) and set to capture images at 5-min intervals. The remaining intact root bundles were placed in a shallow petri dish, and paper towel was gently wrapped around the exposed roots between cameras, to allow for re-saturation. Psychrometers were attached to each plant and set to measure water potential as described above.

The first optical experiment (Supplementary Fig. S2, A and B) was designed to test whether local xylem refilling was observable in partially and completely embolized roots after rewatering directly onto root surfaces. When the first three plants (Supplementary Fig. S2A) reached −3.5 MPa (partial root system embolism, but majority leaf embolism), water was gently dripped directly onto the glass slide within each of the two cameras to capture rehydration of roots in immediate contact with water, while the remaining root bundle re-saturated to allow whole plant rehydration. Plants were covered with damp paper towel and plastic to facilitate rehydration. The next three plants (Supplementary Fig. S2B) were allowed to fully embolize and then were gently cut at −5.0 MPa to ensure no tension remained in the xylem. Water was then dripped directly onto the glass slide within each of the cameras to test for rehydration of fully embolized roots.

The second optical experiment, using three plants for each of the three water potentials (Supplementary Fig. S2, C to E), was intended to determine whether water could be pulled into the embolized roots positioned in the cameras from adjacent saturated roots (indirect rehydration), testing for the recovery of water transport following embolism. Plants with cameras in series along seminal roots were setup as above. Once at the target water potentials of −2 MPa (Supplementary Fig. S2C), −3.5 MPa (Supplementary Fig. S2D), and −5 MPa (Supplementary Fig. S2E) the paper towel between cameras was saturated to ensure the roots between cameras were fully wet, and the remaining root bundle was emersed in water. This supplied abundant water to be pulled into the field of view (<5 cm from the saturation point) of the camera if embolism repair and functional recovery of water transport were to occur, without directly wetting the region within the camera. During all rehydration experiments (excluding Supplementary S2B with cut roots) leaves were covered with damp paper towel and plastic. Cameras continued to capture images for 24 h after rehydration. Finally, with the cameras still attached the roots were cut to induce 100% root xylem embolism.

### Optical image analysis

Image stacks were analyzed in IMAGEJ (NIH) with the OSOV toolbox, using the optical method (Brodribb et al. 2016), see https://www.opensourceov.org/. Firstly, to detect xylem embolism events within each individual root, images in sequence were removed from the previous frame using image subtraction (Brodribb et al. 2016) to reveal any differences in pixels between frames. These pixels, indicating changes in light transmission through rapid air-filling of vessels, were extracted over the sequence of images revealing the spread of total xylem embolism. Pixel changes (as a % of the maximum embolized pixel area) were then plotted against the stem psychrometer data to determine the water potential where embolisms occur.

The image subtraction method was used to detect potential xylem refilling events. The liquid water phase in xylem easily transmits light, while air embolism impedes the transmission of light, resulting in a gray value pixel change between images. The ImageJ OSOV toolbox was utilized to detect differences between images, with the positive pixel values indicating the presence of emboli and the null values indicating the absence of emboli, showing the disappearance of gray-value pixels limited to regions within the root xylem as xylem refilled. Thus, the same software would be used to analyze embolism or refilling through the accrual of positive pixel values to determine embolism, or through the disappearance of positive values to look at refilling. Functionally, 8bit image stacks were reversed so that the image subtraction software (Brodribb et al. 2016) could detect changes in light transmission, and then resulting outputs were returned to the original sequence to look at refilling events.

### Rates of water potential recovery post-rehydration: bare-root, CT, and soil

The removal of roots from soil was hypothesized to be a disruptive process that may cause changes in the function of roots, and thus impact the rate of recovery of water potential within the plant. Similarly, repeat MicroCT scanning of living plant material has been highlighted as having the potential to cause cell death or imped function (Petruzzellis et al. 2018), potentially resulting in altered patterns of root water uptake in the rehydrated plants. To determine the impact of the methods on water potential recovery post-rehydration from −3.5 MPa across treatments, we compared the 12 h rehydration curves in the three MicroCT scanned plants, the six optical vulnerability bare rooted plants, and the three soil rehydrated plants prior to xylem staining, using loess to calculate a mean rehydration rate, with the understanding that significant damage to root function could be visualized through the rehydration rates. The unscanned bare rooted plants showed a marginally faster mean rate of recovery compared with MicroCT scanned plants, but showed complete overlap with the recovery of all repeats (Supplementary Fig. S1). This suggests that although the repeat CT scanning may have some degree of impact on living cell function, the recovery of water potential in the scanned plants at a rate equivalent to unscanned plants indicates that a significant proportion of the CT scanned root system retains the same degree of function as the unscanned plants. Given this complete overlap in the rates of recovery in scanned and unscanned plants, we do not think that the potential damage is enough of a reason to exclude this data. The recovery of bare-rooted plants was much quicker than the in situ soil plants, suggesting that the removal from soil had not impeded the rate of water uptake, and instead inconsistent saturation through the soil was impeding the rate of water uptake despite the pots sitting in standing water (Supplementary Fig. S1). However, these data cannot exclude the possibility that X-ray dose may have affected the cellular processes (RNA integrity and protein synthesis) as shown by Petruzzellis et al. (2018), potentially involved in the active xylem embolism repair process, so that the MicroCT data should still be treated with some caution as a standalone finding.

### Statistical tests

Estimates of the percentage xylem root network dysfunction recorded using xylem staining, optical, and MicroCT methods (note that the optical and MicroCT percentages were sourced from Harrison Day et al. (2023)) over decreasing water potential thresholds were calculated using a two-way ANOVA with unequal sample size. The ANOVA compared the effect of method and water potential on the mean percentage dysfunction of the root network. The interaction term between method and water potential was found to be nonsignificant and was dropped from the model. All statistical tests and data figures were produced using R (R Core Team 2024), all image post-processing was produced using Inkscape (Inkscape 2021).

## Supplementary Material

kiae407_Supplementary_Data

## Data Availability

The data that support the findings of this study are available from the corresponding author upon reasonable request.

## Supplementary data

The following materials are available in the online version of this article.

**Supplementary Figure S1.** Rehydration kinetics of wheat plant re-saturated via roots over 12 h overnight following optical experiments (OV), MicroCT scanning (CT), and soil resaturation (soilsat).

**Supplementary Figure S2.** Direct and indirect rehydration of embolized wheat roots over water stress treatments using image capture.

**Supplementary Figure S3.** Results summary of the Optical Method treatments visualizing embolism/refilling.

**Supplementary Figure S4.** Xylem cavitation and rehydration of wheat roots dried to −2.0, −3.5, and −5.0 MPa (*n* = 3 plants per treatment, three to four roots per plant in two regions).

**Supplementary Video S1.** Refilling of cut, fully cavitated roots could be demonstrated using the optical method. Left panel shows the raw colour images of root rehydration in 3 minute intervals, taking 45 minutes to fully refill roots. Right panel shows the image differences produced by the image subtraction method able to detect refilling in these circumstances.

## Dive Curated Terms

The following phenotypic, genotypic, and functional terms are of significance to the work described in this paper:

xylem AmiGo: PO:0005352

dye CHEBI: CHEBI:37958

root AmiGo: PO:0009005

water CHEBI: CHEBI:15377

whole plant AmiGo: PO:0000003
