# Supplementary material for: A one-way ticket: Wheat roots do not functionally refill xylem emboli following rehydration
Source: Plant Physiol. 2024 Sep 19;196(4):2362–73. doi: 10.1093/plphys/kiae407 (PMC11638109; doi:10.1093/plphys/kiae407)
Supplement: kiae407_Supplementary_Data [file kiae407_supplementary_data.zip › PP2024-RA-00313DR1_Supplemental_Figures_1_4.docx]

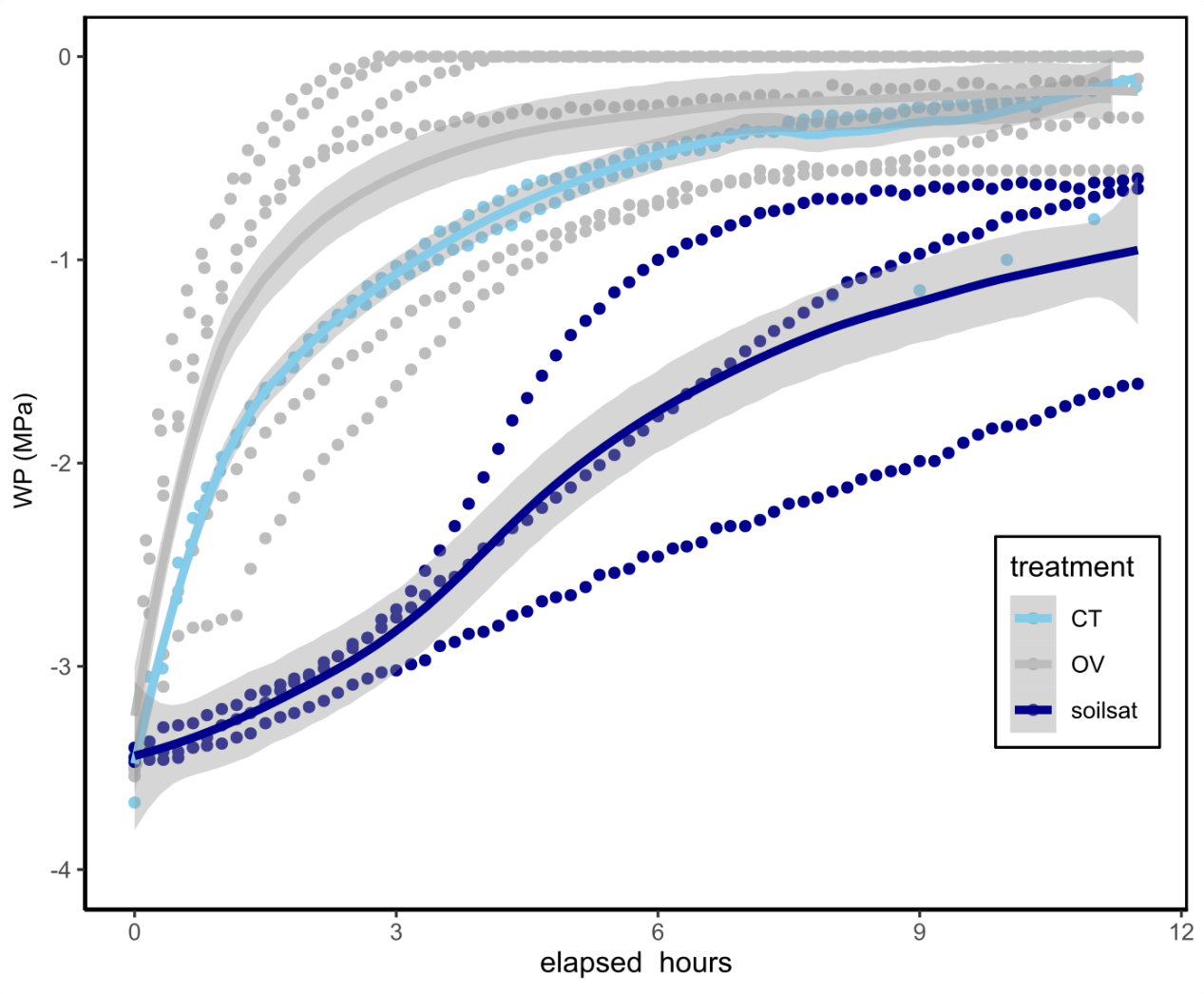


Supplementary Figure S1: Rehydration kinetics of wheat plant re-saturated via roots over 12 hours overnight following optical experiments (OV), MicroCT scanning (CT) and soil resaturation (soilsat). Solid lines show mean rates of recovery per method (loess). OV and CT rates of recovery showed overlap in the rates of recovery within individual plants, however, the mean rates of recovery were slower in CT scanned plants, indicating some level of damage. Soil saturated plants were slower than other methods despite sitting in abundant standing water, indicating some barrier to the movement of water through soil to the root system. WP = water potential (MPa).


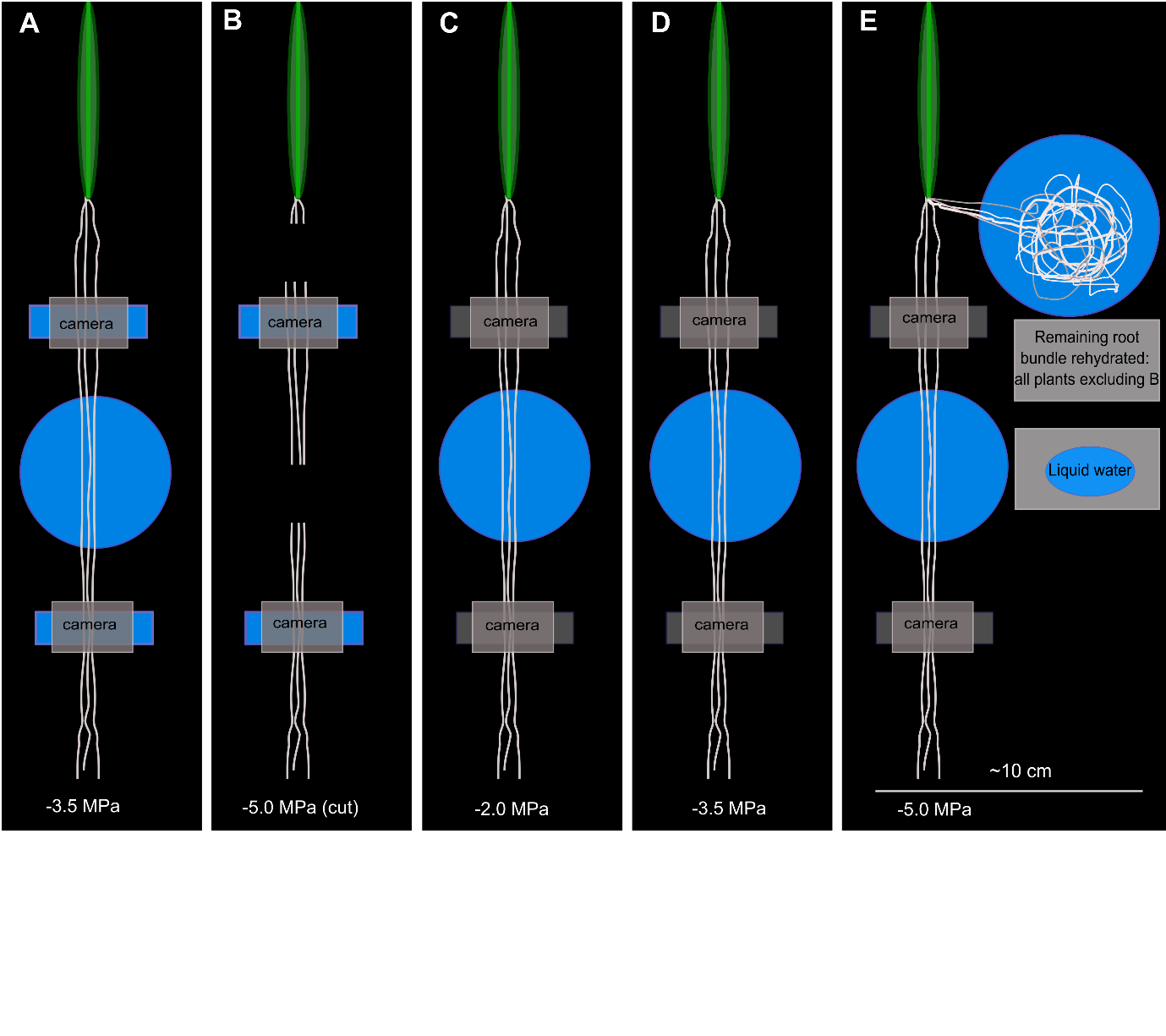


Supplementary Figure S2: Direct and indirect rehydration of embolized wheat roots over water stress treatments using image capture. Design of Optical Method treatments (A-E) showing position of cameras along the length of intact seminal roots of wheat. Roots (white) and the location of rehydration with liquid water (blue) within or external to camera units (grey) containing roots between glass slides (rectangular regions within cameras). Thresholds of water stress (MPa) indicated at the bottom of each treatment facet (A-E). A and B show rehydration directly onto the region of observation. C-E show rehydration adjacent to the region of observation. N=3 plants were used per treatment (A-E) (15 plants total). Intact roots shown as continuous white lines (A, C-E). Cut roots indicated as discontinuous lines (B).


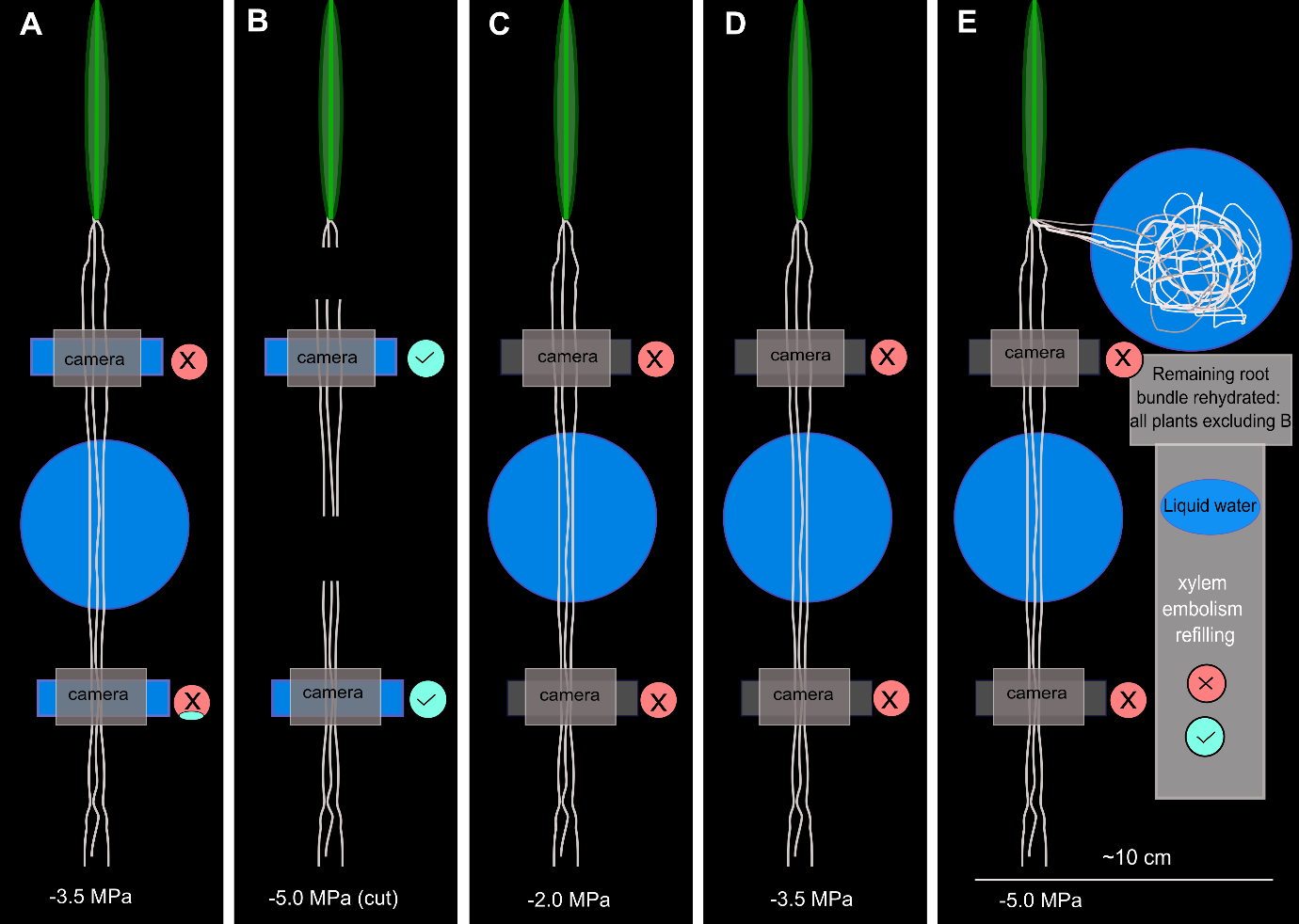


Supplementary Figure S3: Results summary of the Optical Method treatments visualising embolism/refilling. Xylem embolism refilling could not be demonstrated in the roots of wheat when saturated directly (water = blue) onto the localised region of interest within the camera at -3.5, with the exception of two root segments out of 22 (A) (failure to refill = red X). Xylem embolism refilling could be demonstrated in the fully embolized, cut roots when saturated directly onto the localised region of interest within the camera at -5.0 MPa (B). Xylem embolism refilling could not be demonstrated when rewatered adjacent to regions of root within cameras at -2.0 MPa (C), -3.5 MPa (D) and -5.0 MPa (E) (refilling observed = green ✓).


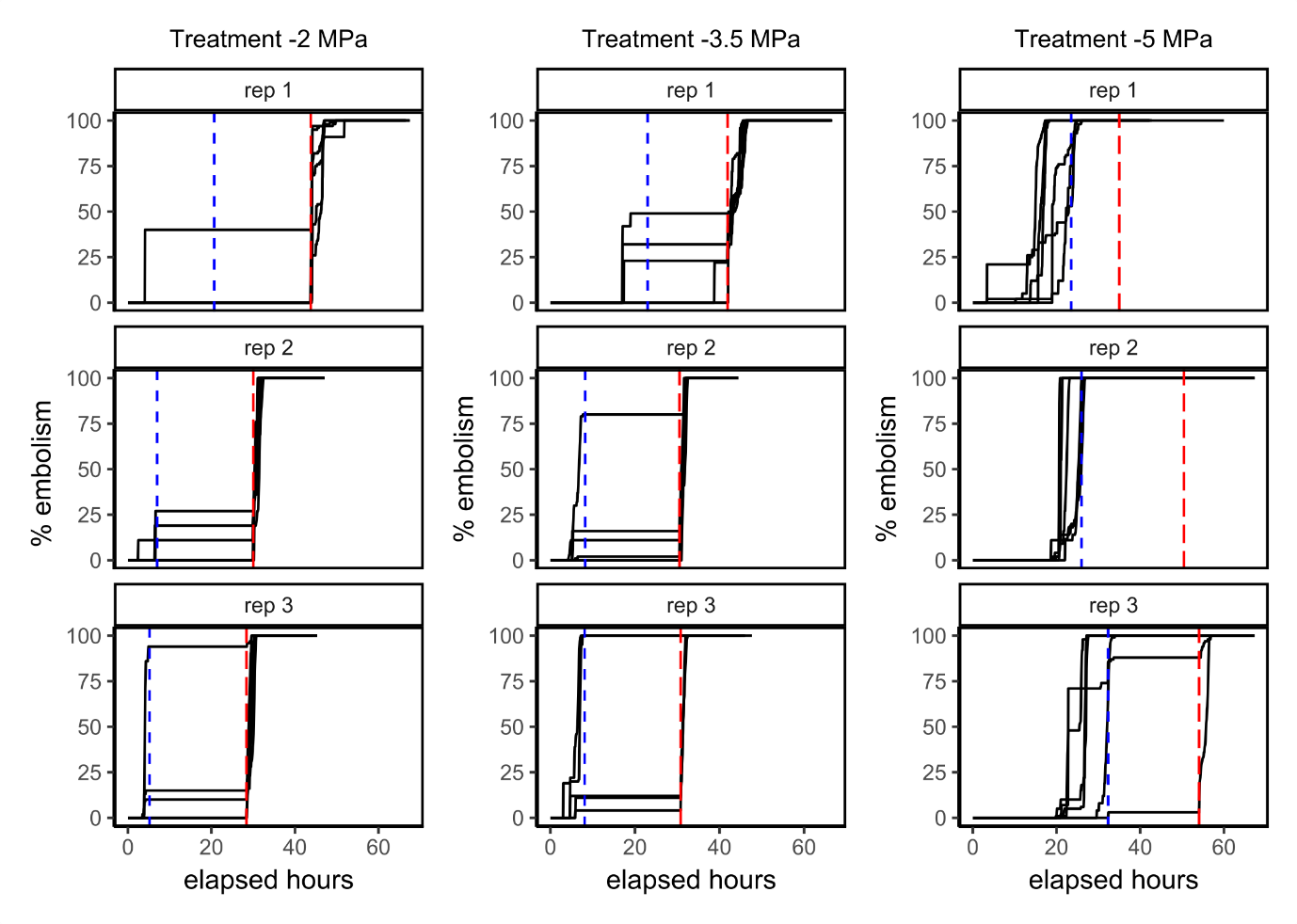


Supplementary Figure S4: Xylem cavitation and rehydration of wheat roots dried to -2.0 MPa, -3.5 MPa, and -5.0 MPa (n=3 plants per treatment, 3-4 roots per plant in two regions). Vulnerability curves constructed from two cameras placed longitudinally along the root section. Xylem embolism refilling could not be demonstrated in the roots of wheat when saturated adjacent to regions of root within cameras.
