# Supplementary material for: A one-way ticket: Wheat roots do not functionally refill xylem emboli following rehydration
Source: Plant Physiol. 2024 Sep 19;196(4):2362–73. doi: 10.1093/plphys/kiae407 (PMC11638109; doi:10.1093/plphys/kiae407)
Supplement: kiae407_Supplementary_Data [file kiae407_supplementary_data.zip › Supplementary Video Legend.docx]

**Supplementary Video Legend**

Supplementary Video S1: Refilling of cut, fully cavitated roots could be demonstrated using the optical method. Left panel shows the raw colour images of root rehydration in 3 minute intervals, taking 45 minutes to fully refill roots. Right panel shows the image differences produced by the image subtraction method able to detect refilling in these circumstances.
