# Supplementary figures and images for: A one-way ticket: Wheat roots do not functionally refill xylem emboli following rehydration
Source: Plant Physiol. 2024 Sep 19;196(4):2362–73. doi: 10.1093/plphys/kiae407 (PMC11638109; doi:10.1093/plphys/kiae407)

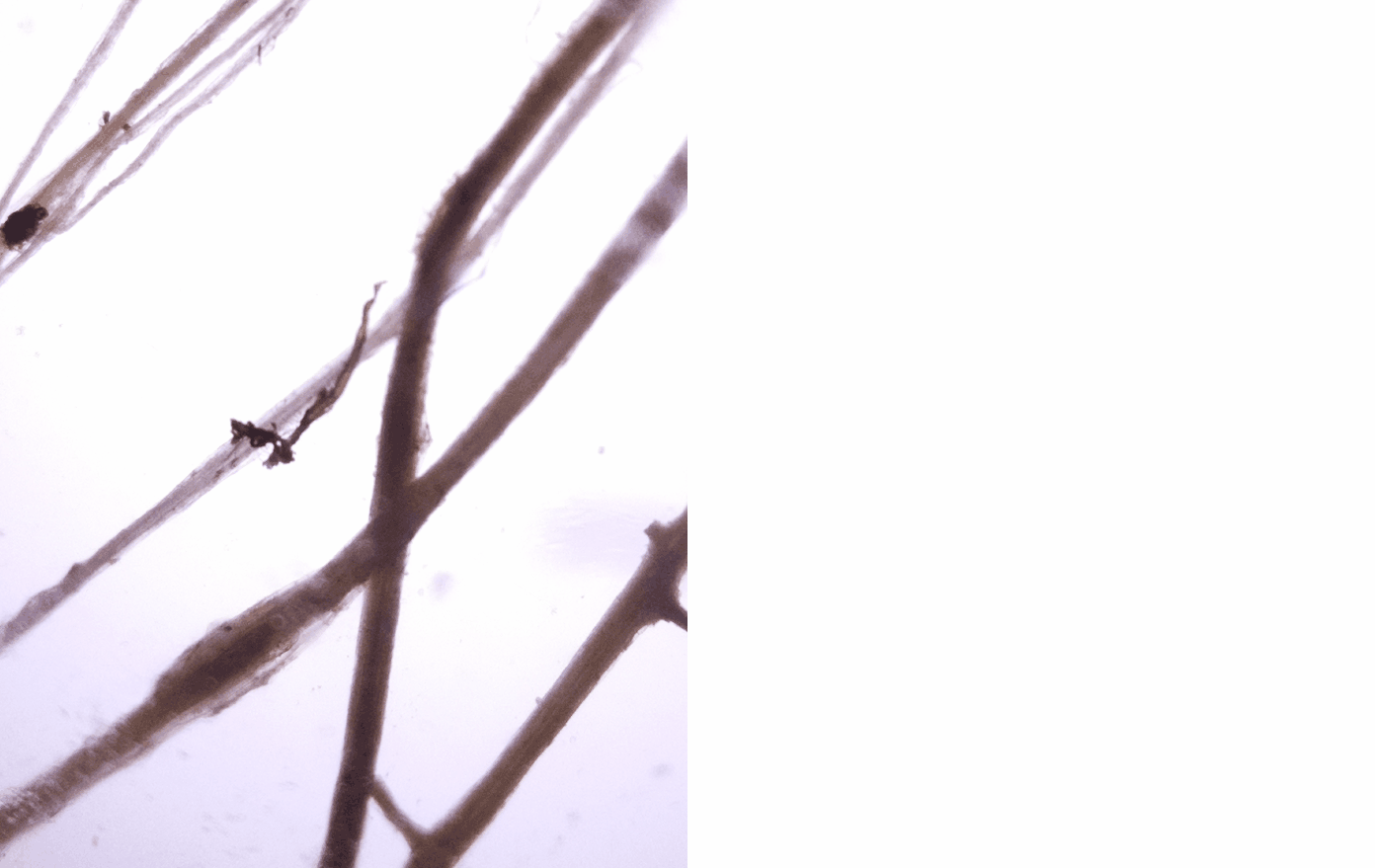

Supplement: kiae407_Supplementary_Data [file kiae407_supplementary_data.zip › supplementary video.gif]
