# Supplementary material for: A one-way ticket: Wheat roots do not functionally refill xylem emboli following rehydration
Source: Plant Physiol. 2024 Sep 19;196(4):2362–73. doi: 10.1093/plphys/kiae407 (PMC11638109; doi:10.1093/plphys/kiae407)
Supplement: kiae407_Supplementary_Data [file PP2024-RA-00313DR1_Merged_Decision_Summary_PDF.pdf › PP2024-RA-00313DR1_Merged_Decision_Summary_PDF.pdf]

Plant Physiology®

A one-way ticket: Can wheat roots functionally refill xylem emboli following rehydration?

Beatrice Harrison Day, Kate Johnson, Vanessa Tonet, Ibrahim Bourbia, Christopher Blackman, and Timothy Brodribb

Review Timeline:

Submission Date:	06-Nov-2023
Editorial Decision:	07-Dec-2023
Resubmission Received:	06-May-2024
Editorial Decision:	29-May-2024
Revision Received:	18-Jun-2024
Accepted:	21-Jun-2024

06-Dec-2023

Prof. Tim J Brodribb
University of Tasmania
Hobart, TAS
Australia

RE: A one-way ticket: Can wheat roots functionally refill emboli after xylem cavitation?

Dear Dr. Tim Brodribb:

Thank you for giving us the opportunity to review your manuscript for publication in the Plant Physiology. The reviewers have found the manuscript of interest, but have raised a number of concerns that will require your attention. As these concerns are of some gravity and will entail substantial changes to the manuscript, you are likely to need additional time to address them. I must therefore reject the manuscript in its present form. Nonetheless, if you are able to address the points raised in review, I would welcome your resubmission of a manuscript that incorporates your responses.

In particular, I direct your attention to the following comments for consideration in a resubmitted manuscript.

1. Both reviewers raised the issue of the high likelihood of lethality of the prolonged and repeated microCT scanning, unless a test was or could be made to demonstrate the viability and sustained survival of specific root tissue after repeated scanning this data should removed from the manuscript.
2. One reviewer raised the need to increase the number of replicates for the optical assessment of refilling, a point I am inclined to agree with, particularly if these could be designed in such a way as to assess potential refilling in intact plants with roots still in soil and not unpotted and roots washed-which was also raised as an issue.
3. One question highlighted the absence of a positive control with regards to whether refilling in wheat could ever be detected optically. This important data is hidden in the supplemental and should be in the main text.

Please note that any resubmission will be treated as a new entry to the system and is likely to go out for review.

If you do have any queries or concerns, you are welcome to contact me in the first instance.

Thank you again for allowing Plant Physiology to review your work. We look forward to hearing from you soon.

Sincerely,

Editor, Plant Physiology

-- Reviewer comments:
Reviewer #1 (Comments for the Author):

see pdf document

Reviewer #2 (Comments for the Author):

Harrison Day and co-authors have performed some drought-rewatering lab experiments, using in vivo (Micro-CT and optical OV method) and ex vivo (safranine staining) techniques to show that wheat roots are not able to refill embolized metaxylem upon rehydration after drought, imposed at different water potential levels reached through bench dehydration of bare rooted plants in the lab. The authors measured water potentials during drought progression with a stem psychrometer mounted on the peduncle of the plant and rewatered locally the plants with different methods, depending on the embolism detection technique.
The manuscript is generally clear and well written. The experiment has been overall well planned and performed and the overall outcome seems reasonable. However, there are some methodological and statistical (lack of replicates) issues regarding the two in vivo methods that would need further clarification and testing.

Issue about the optical method. For each of the 6 rehydration treatments (shown in the nice fig S2)it seems there was only one replicate plant. Thus, the experiment does not have the statistical solidity required.

Other comment on the OV substraction method: that's an interesting approach. However, "active" refilling of xylem vessels

would be a slow, progressive process, much slower than embolism, which instead should be almost instantaneous, and likely slower than refilling by capillarity (shown in the plant B). Could maybe substraction of images taken at 3 min time lag not be able to detect an active refilling phenomenon, given that this should happen in longer times? Moreover, Film 1 shows the rehydration and refilling by capillarity, which was shown in the plant B. Have the substacks shown on the right been obtained with the "reverse image" approach? Please specify. Moreover, it is not completely clear to me how the reverse substraction works in order to quantify the refilling and show the occurrence of the process. For clarity, the authors could provide a figure where a sequence of reverse raw images and the related substacks of refilling are shown.

Issue about X-Ray Micro-CT. Ll 214-226: Scanning procedure at Syncrothron. For each plant, the same root bundle was scanned with X-ray (at 40 keV, for 11 min every scan) at the same region 3 consecutive times, i.e. at plant full hydration, at dehydration (at plant WP= -3.5 MPa) and after 12 h rehydration. The authors did not verify if the X-ray dose received by the root could damage cell functionality, and therefore the possibility of refilling. Multiple scans can alterate cell membranes, RNA integrity etc, thus inhibiting several cellular processes (Petruzzellis et al. 2018, New Phytologist). This possible artefact could had been avoided by scanning different plants just once at the different hydration steps (i.e. a total of 9 plants).

Other comments:
L. 295: where was this information taken from? Is there any information on wheat root regrowth time after rehydration? Please provide it. It should also depend on the drought level reached before rehydration.
L. 265: "was" should replace "were", as it refers to "group"
L 432: was should replace were

Response to editor:

Dear monitoring editor Dr. .

Thank you for your invaluable review of our manuscript PP2023-RA-01742 entitled "A one-way ticket: Can wheat roots functionally refill emboli after xylem embolism?" in Plant Physiology. We are very encouraged that the reviewers found the manuscript of interest and we are appreciative of their time and dedication in making their helpful suggestions. We are particularly grateful for the specific direction of reviewer 2 in their feedback.

We are appreciative of your consideration of the time involved in achieving the suggestions of reviewers. The resubmitted manuscript contains the changes requested, with a particular focus of a full repeat of the Optical Vulnerability experiments. As per the suggestions of the reviewers and managing editor, we have chosen to repeat all of the OV experiments, taking the sample size from n=5 (1 plant per experimental treatment) to now n=15 (3 plants per treatment) for this section of the work. While we agree that visualising embolism and possible refilling in soil would be ideal, the application of cameras and the controlled rehydration of these roots requires bare roots. However our application of a novel dye technique confirmed that the root xylem behaviour observed in soil was identical to that observed in soil-free roots. We hope that the newly repeated optical experiments help support our hypotheses, and now meet the expectations of reviewers and editor. Here, we hope that the larger bare-root OV sample size, in combination with the paired in-soil and bare-rooted xylem staining experiments, now provide a more convincing argument. Thank you for your advice that we include the positive control data in the main manuscript. We have now done so. Based on your suggestion, and given the informative nature of the repeat experiments, we have made a new figure showing direct rehydration at the key water potential of -3.5 MPa, and the positive control in fully embolised roots. This is now presented as Fig. 5 in the main manuscript. The original figure 5, which included indirect rehydration data, has been updated to include the new replicates and moved to the supplement. The specifics of these changes can be found in greater detail in the response to reviewer 2 below.

Regarding the MicroCT data, we agree that the repeat scanning of living plant material is likely to introduce some degree of damage. We have carefully changed the wording to ensure there is qualifying statements surrounding the equating of this data with undamaged plants. We propose the following evidence in support of including the CT data in our paper. The rate of uptake through the living tissues of scanned roots is the process most likely to by damaged by x-rays. We suggest that comparing the rate of water uptake between scanned and unscanned plants should give a meaningful indication of the level of damage introduced by scanning. We have made a new figure showing the mean rates of water potential recovery post rehydration in the three CT scanned plants, and the now six replicates from the OV tests in plants pushed to the same threshold of -3.5 MPa, as well as the recovery of the soil re-saturated plants pushed the same water potential. The mean rehydration curves of the CT scanned plants were slightly slower than the mean of unscanned plants, supporting your concerns. However, the margin was small, and two of the unscanned plants had rehydration rates that were slower than the CT, showing complete overlap between the scanned and unscanned plants. This data has been included as Fig. S1 and is available for reviewers and readers now. Given this complete overlap in the rates of recovery in scanned and unscanned plants, indicating that a significant proportion of the CT scanned root system retains the same degree of function as the unscanned plants we do not think that the potential damage is enough of a reason to exclude this data. We have chosen to keep the figure in the main

document but have added significant written qualification to explicitly address the potential for error in the methods, results and discussion.

Finally, given a review comment on the interchangeable use of embolism and cavitation, we have changed the title of the manuscript from “A one-way ticket: Can wheat roots functionally refill emboli after xylem embolism” to **“**A one-way ticket: Can wheat roots functionally refill xylem emboli following rehydration?” We hope this meets your approval, however, we will happily return it to the original title if you have concerns.

Please find below a comprehensive list of all changes made in response to advice by reviewers.

Response to reviewer 1:

Thank you for your valuable feedback on our manuscript. Please find below a detailed list of all the changes we have made in response to your comments (listed in bold). Corresponding changes may be found in the “combined copy of manuscript with track changes including figures” document. All line numbers correspond to this document.

Although there is clear evidence that the wheat plants studied did not show any refilling, it is unclear to me why the authors did not consider positive xylem pressure in more detail because we cannot exclude that refilling could be possible when positive xylem pressure would occur, as has been shown for instance for maize (Gleason et al. 2017). We know that wheat (like probably any other Poaceae species) is able to develop positive xylem pressure and guttation. This process may not result in embolism refilling, but perhaps only in rehydration, and may facilitate growth at night. Also, why did positive xylem pressure not occur in the plants studied? Uprooting plants is a rather dramatic, unnatural process, and it is also known that the development of positive pressure may only work under particular environmental conditions that did not occur in the experiments conducted. Note also that the term root pressure may not be appropriate for wheat (and any other grass) because there is no evidence that positive xylem pressure finds it origin in its fibrous roots.

Thank you for suggestion. We agree this was missing from the manuscript and we hope now to give a better contextualisation to this process. Given your suggestion, we have included reference to positive pressure and leaf guttation in the introduction on line 112-113. We have specifically addressed your suggestion that xylem refilling may occur in plants generating positive pressure, which ours did not, in the discussion on line 548-559. We agree that this deserves a more nuanced focus within the manuscript. We agree that performing these experiments on bare rooted plants is dramatic, unnatural process, as you highlight, but we note that the results of root dye experiments (where roots remained undamaged in soil) were completely consistent with soil-free root behaviour. We agree that this potential lack of biological reality should be described. We have highlighted this point in the methods on line 387-409, along with the rates of recovery for CT scanned plants.

The broad spread in vulnerability of wheat roots is at least remarkable and needs an explanation (e.g. line 221-522). Why is there a high carbon investment in some roots that are capable of surviving until the death of the plant (Line 528-530)?

Thank you for highlighting these points. This data is available in an earlier paper (as you identify), but we agree that these comments are integral to the understanding of this manuscript. We have included a more detailed explanation of the meaning of these comments in a rewritten paragraph of the discussion on lines 561-587. We have also included a statement on line 142-146 of the introduction to provide some context.

Are these super-roots different from normal roots in some anatomical or morphological way? The reader is reminded here to look at earlier papers by the authors, but an explanation why xylem conduits show this large variation is not convincing.

Thank you for your question. The xylem anatomy is not the focus of this paper, however, the behaviour of roots measured here was entirely consistent with previous work, and with continuing work on other species. It was also consistent across methods, and in terms of the rehydration characteristics of plants after drought. In direct answer to this question, however: these are not super roots. These roots are not morphologically different from "normal roots". There is simply a range in root sizes and placements throughout the root system, with tapering of root sizes and vessel sizes getting progressively smaller towards the periphery of the root network. We chose to examine the largest three-four roots for ease of image capture using the optical methods. All other root classes empty into this cohort, and these feed directly to above-ground tissues, making embolism repair in these roots the key determinant for water transport to the rest of the plant. There is no clear anatomical division between these root types and the rest of the root network: these are simply the largest. For dye rehydration tests, the largest 50 roots make up the majority of the root network. Larger roots tended to contain larger xylem of a greater number, while smaller roots contained fewer and smaller xylem. Here, only the finest, tiny roots and root hairs are excluded and these are likely still growing and do not yet have a functional xylem connection. Data from (Harrison Day et al., 2023) shows that these tiniest roots have few, very small xylem. As safranin is a physical particulate, we chose to exclude the tiniest roots under the assumption that these particles may become occluded in pit membranes. We have not excluded a significant proportion of the root network here, include most of the root network, only excluding the finest roots that we have been unable to capture embolism in with other methods. We have included extra justification in our methods on lines 314-318.

Are these observations based on measurement artefacts based on inaccurate water potential estimations…

In terms of the accuracy of water potential measurements, we have addressed this point in Fig. S2 of our previous works (Harrison Day et al., 2023) to greater detail. In short, it was raised by reviewers in previous publications that the rate of transpiration from roots may have an impact in the broad distribution in vulnerabilities. To address this, we conducted a repeat experiment with four cameras spread across 50 roots of a single plant, with the entire root system with cameras enclosed in sealed bags with damp paper, to halt the root transpiration, so that all water potential decline was driven by transpiration of the above-ground tissues. The broad array in vulnerabilities remained the same, and was also validated by MicroCT, indicating that this large spread in vulnerabilities was a valid pattern and not measurement error in water potential. We are confident, and have shown to the full extent of previous

reviewers requests, that this variation in vulnerability data is not an artifact of measurement protocols.

…or is this variation related to different developmental stages (or age) of the fibrous roots, or perhaps by earlier embolism events in protoxylem conduits that cannot be observed with the optical method?

The measurements on vulnerability referred to in the earlier work were determined through Optical Methods and though MicroCT. The measurements on xylem anatomy were determined through microscopy. The variation relates to the size of roots and their position within the root system. The peripheral parts of the root system were more vulnerable than those regions close to the root collar. This details are now clearly specified in the discussion (561-587) We believe that both protoxylem and metaxylem events may be captured by the optical methods. We have unpublished data that shows that the mean number of cavitation events picked up by OV in wheat roots is 13.5 (in the same root sizes examined here). The greatest number metaxylem observed in any of the roots from light microscopy was 6, indicating that protoxylem cavitation can be clearly observed using the optical method in these roots.

It sounds reasonable to assume that peduncle water potentials can be used as an estimate of root water potential. However, this should be tested. The Shardakov method, for instance, could be applied to root tissue to estimate water potential values.

Thank you for your suggestion. We would like to refer you to the above comments regarding previously published work validating the use of stem water potential in roots.

Line 122-123: these are not relevant papers related to freezing artefacts with cryo-SEM. See for instance Umebayashi et al. 2016 (Trees) or Yazaki et al. 2019 m(J. Vis. Exp.).

Thank you for highlighting that the references used were not appropriate. We have used your suggestions. These have been updated on line 122.

MicroCT scanning of 11 minutes is very likely to kill the poorly lignified wheat roots, which could also be an explanation why no positive xylem pressure was observed in the microCT samples.

Thank you for your comment. The managing editor, as well as the second reviewer, also highlighted that the CT scanning of living tissues is likely to result in some degree of cell death. This possibility was what led us to employing two other techniques to verify these observations. The agreement of all techniques, including the dye method which characterizes root water uptake in soil bound roots, give us confidence that the observations from the CT are worthy of presentation. We think there is some merit to including the CT data, but we have carefully changed the wording to ensure there is qualifying statements surrounding the equating of this data with undamaged plants in the methods, results and discussion. We propose the following evidence in support of keeping the CT data included. Given that xylem are dead when functional, the CT scanning should have little effect on the capacity for water transport within the root system. The rate of uptake through the living tissues scanned roots, however, is the process most likely to by damaged by x-rays (as you rightly highlight). We suggest that comparing the rate of water uptake between scanned and unscanned plants should give a meaningful indication of the level of damage introduced by scanning. We have

made a new figure showing the mean rates of water potential recovery post rehydration in the three CT scanned plants, and the now six replicates from the OV tests in plants pushed to the same threshold of -3.5 MPa, as well as the recovery of the soil re-saturated plants pushed the same water potential. The mean rehydration curves of the CT scanned plants were indeed slightly slower than the mean of unscanned plants, supporting your concerns. However, the margin was small, and two of the unscanned plants had rehydration rates that were slower than the CT, showing complete overlap between the scanned and unscanned plants. This data has been included as Fig. S1 and is available for reviewers and readers now. Given this complete overlap in the rates of recovery in scanned and unscanned plants, we do not think that the potential damage is enough of a reason to exclude this data, and have chosen to keep the figure in the main document, but with significant written qualification. While we understand the CT will introduce a degree of damage, the recovery of water potential in the scanned plants at a rate equivalent to unscanned plants indicates that a significant proportion of the CT scanned root system retains the same degree of function as the unscanned plants. These details have been included in the methods as a dedicated segment in lines 388-409.

Specifically regarding your comment on the failure of CT scanned plants to generate positive pressure: none of our plants observed produced positive pressure from the evidence of psychrometers. This was no different in the CT scanned plants, the bare-rooted plants used in the optical methods, or in the completely intact plants in soil used for the root staining. This raises a number of additional questions regarding whether the loss of root function through xylem cavitation impacts the generation of root pressure, however, as there was no function difference between CT scanned, unscanned plants, and plants with roots in soil, there is no reason to exclude this CT data. As with your earlier suggestion, we have included a new segment in the discussion on the absence of root pressure on lines 548-559.

Line 147: this staining method is not novel and has been applied in earlier papers.

Thank you for identifying this. Although active xylem staining using safranin has been used in previous studies, we believe it has not been used in this manner to investigate root functional recovery post embolism. However, given your concerns we have de-emphasised the novelty through the manuscript.

The xylem staining is described to demonstrate "xylem function", for instance in line 415 and the caption of Fig. 2. However, the fact that no dye is taken up does not mean that there is no transport because of embolised conduits. Root hairs may have dried up, or the endodermis may not be able to transport any symplastic water. In this way, it is more accurate to talk about "root network dysfunction", as done in line 437 and the caption of Fig. 3. Yet, "% root network dysfunction" is used in figure 3 for two different processes, namely embolism (MicroCT, optical method), and root network dysfunction (xylem staining). It is rather misleading to include both processes in the Y-axis of this graph.

Thank you for identifying that this is unclear. Given your suggestion we have used "root network dysfunction" throughout the manuscript now. This has been updated in lines 131. We agree that the figure 3 includes comparison of multiple processes. For simplicity we have kept the Y axis intact, as a means of displaying the different treatments together, as the % root network embolism is directly equivalent to the number of roots that failed to stain.

What is the difference between embolism and cavitation? Both seem to be used as synonyms, along with "emboli". Please define these clearly. As far as embolism formation is understood, there is no evidence that this process results from cavitation or the creation of a void in a liquid.

Thank you for identifying this inconsistency. We have changed all references to embolism. Although cavitation is valid for describing the initiation of air filling, we have chosen to use embolism or emboli (plural) in the interests of simplicity. Given your concerns, we have changed the title to the simplified "A one-way ticket: Can wheat roots functionally refill xylem emboli following rehydration?"

Response to reviewer 2

Thank you for your clear and informative review of our manuscript. We are very appreciative of the time taken to improve our work, and are encouraged by your interest in the subject matter. We are very glad that you find the outcomes reasonable and we hope the following changes reinforce this opinion. We agree that our manuscript was previously lacking in methodological and statistical replication that detracted from the conclusions. Thank you for giving us the opportunity to correct these. We have fully repeated the Optical experiments to increase the statistical power. Corresponding changes may be found in the "combined copy of manuscript with track changes including figures" document. All line numbers correspond to this document.

Please find below a detailed list of all the changes we have made in response to your comments (listed in bold).

Issue about the optical method. For each of the 6 rehydration treatments (shown in the nice fig S2) it seems there was only one replicate plant. Thus, the experiment does not have the statistical solidity required.

Thank you for highlighting this. In response, we grew new batch of the same genotype and have fully rerun these experiments. The initial OV methods had 5 plants (1 per treatment). We now show 15 plants (3 per treatment, with multiple roots per plant). All results remain the same, showing roots fail to refill through indirect rehydration in all cases, and fail to refill when rehydrated directly at partial embolism. The only exception from the repeat experiments were 2 individual root segments out of the total 22 in the -3.5 MPa that showed some evidence of refilling. We have explicitly addressed this in the manuscript as a percentage. We hope that, given your suggestions, we now present a much more detailed and nuanced interpretation of this story and are grateful to have been given the opportunity to correct our error. These additional reps can be found in figure 5 and in the supplementary figures. The details have been updated in the methods.

We have also chosen to change the following aspects in the methods and results: In our first presentation of the OV data, we used three cameras placed along the length of the roots. We have determined that it is not informative to have all three cameras, as this just increases the degree of pseudo-replication and resampling of the same roots. We have reduced this to two cameras per plant in the repeat experiments (using the data from the two cameras of the initial reps closest to the root collar and excluding the final, furthest camera) to reduce pseudo-replication. We chose to keep two cameras to ensure that we could have lengths of root with water saturated in between effectively. The cameras contain multiple root segments that are

bundled together in the data, but we now have 3 plants per treatment, increasing the sample size to ~22 root segments per treatment. We hope this is a more rigorous method compared to our initial experiment.

We have also given considerable thought to whether indirect rehydration is likely to functionally occur in a biological reality. Given the slower rate of recovery in the soil-saturated plant, we believe that it is common for parts of the root system to be saturated and parts to not be in direct contact with liquid water, as the experiment reflects. This data displaying the rate of recovery is now available in Fig. S1. We have, however, decided that examining the direct rehydration of root tissues is more interesting for the reader. For this reason, we have moved the direct rehydration data to the main manuscript, and the indirect rehydration data to the supplement. We hope that this change is acceptable, especially given your below comment about positive controls. Please find these data displayed as Figure 5, and supplementary figures 3 and 4 now. If you should disagree with the change, we will happily return it to the main manuscript.

Other comment on the OV substraction method: that's an interesting approach. However, "active" refilling of xylem vessels would be a slow, progressive process, much slower than embolism, which instead should be almost instantaneous, and likely slower than refilling by capillarity (shown in the plant B). Could maybe substraction of images taken at 3 min time lag not be able to detect an active refilling phenomenon, given that this should happen in longer times? Moreover, Film 1 shows the rehydration and refilling by capillarity, which was shown in the plant B. Have the substacks shown on the right been obtained with the "reverse image" approach? Please specify. Moreover, it is not completely clear to me how the reverse substraction works in order to quantify the refilling and show the occurrence of the process. For clarity, the authors could provide a figure where a sequence of reverse raw images and the related substacks of refilling are shown.

Thank you for highlighting that this method was unclear. We have improved our methods to specify how this was achieved on lines 375-385. We agree that embolism occurs very rapidly, and refilling occurs on a unknown timescale. Although this method was experimental, we are confident in the success. In the rehydrated plants from -3.5 MPa, the embolised regions remain visible when saturated. We have included an example in the new figure 5 for your interest (subset facets C-E). Given that we were consistently able to see refilling in the -5MPa cut roots, we are confident that we are not overlooking potential refilling but are able to detect it.

Issue about X-Ray Micro-CT. Ll 214-226: Scanning procedure at Syncrothron. For each plant, the same root bundle was scanned with X-ray (at 40 keV, for 11 min every scan) at the same region 3 consecutive times, i.e. at plant full hydration, at dehydration (at plant WP= -3.5 MPa) and after 12 h rehydration. The authors did not verify if the X-ray dose received by the root could damage cell functionality, and therefore the possibility of refilling. Multiple scans can alterate cell membranes, RNA integrity etc, thus inhibiting several cellular processes (Petruzzellis et al. 2018, New Phytologist). This possible artefact could had been avoided by scanning different plants just once at the different hydration steps (i.e. a total of 9 plants).

Thank you for highlighting this issue with the CT scanning of living material. Your concerns are entirely valid, and while we would like to have the opportunity to rescan new plants, this

is not an option in this case. Please see the detailed above response to reviewer 1 (lines 178-220 of this document) for full details of the careful changes in justification, plus additional supporting data. We hope the addition of the rehydration rates in scanned and unscanned plants (showing complete overlap in the rate of recovery) help alleviate concerns about the x-ray damage to living cell layers. We have changed the wording throughout the manuscript to explicitly acknowledge that this method may change the normal function of these tissues. We hope these changes meet your expectations.

L. 295: where was this information taken from? Is there any information on wheat root regrowth time after rehydration? Please provide it. It should also depend on the drought level reached before rehydration.

Thank you for identifying this inconclusive statement. The specifics of this regrowth time from different degrees of drought are not available, but we have included additional reference material on the turnover times for grass species roots, which range from days to weeks (now line 297).

L. 265: "was" should replace "were", as it refers to "group"

This has been corrected.

L 432: was should replace were

This has been corrected.

References:

GLEASON, S. M., WIGGANS, D. R., BLISS, C. A., YOUNG, J. S., COOPER, M., WILLI, K. R. & COMAS, L. H. 2017. Embolized stems recover overnight in Zea mays: the role of soil water, root pressure, and nighttime transpiration. *Frontiers in Plant Science,* 8**,** 662.

HARRISON DAY, B. L., JOHNSON, K. M., TONET, V., BOURBIA, I., BLACKMAN, C. & BRODRIBB, T. J. 2023. The root of the problem: diverse vulnerability to xylem cavitation found within the root system of wheat plants. *New Phytologist,* 239**,** 1239-1252.

PETRUZZELLIS, F., PAGLIARANI, C., SAVI, T., LOSSO, A., CAVALLETTO, S., TROMBA, G., DULLIN, C., BÄR, A., GANTHALER, A. & MIOTTO, A. 2018. The pitfalls of in vivo imaging techniques: evidence for cellular damage caused by synchrotron X-ray computed micro-tomography. *New Phytologist,* 220**,** 104-110.

29-May-2024

Ms. Beatrice Linda Harrison Day
University of Tasmania
School of Natural Sciences
Hobart, TAS 7000
Australia

RE: A one-way ticket: can wheat roots functionally refill xylem emboli following rehydration?

Dear Dr. Beatrice Harrison Day:

We are pleased to accept your manuscript for publication in the Plant Physiology. This acceptance is contingent on revision based on your addressing the minor couple of comments from the reviewers, at your discretion.

Please highlight all changes and include a detailed annotation to changes to the text, with line numbers, and noting your responses to the comments.

To submit your revised manuscript, click:

Link Not Available

If you cannot return the revised manuscript within 8 weeks of receipt, please let us know. Otherwise, we will assume that you have elected not to revise the manuscript and are withdrawing it.

Thank you for allowing us to review your work. We look forward to hearing from you soon.

Sincerely,

Editor, Plant Physiology

-- Reviewer comments:
Reviewer #1 (Comments for the Author):

I am pleased with the modifications made to the revised version of this manuscript. The authors have interpreted more accurately their results, and the evidence that refilling does not occur is convincing.
I would like to make one final comment: the amount of drought stress applied is really important, and therefore three treatment are compared in this study. However, no information about the drought deficit is given in the abstract, which makes it hard for readers to interpret the findings based on the abstract only. Please mention at least the -3.5 MPa drought stress level in the abstract, which really seems to be represent extreme drought, and not the drought experienced by wheat plants on an average hot summer day.

Reviewer #2 (Comments for the Author):

I have reviewed the previous version of the manuscript. The authors in this version have performed additional experiments in order to deal with the lack of replicates in the optical method approach, that was one of my major concerns. Now the results are more solid from a statistical point of view.
Also the description of the substraction method to detect embolism recovery is more clear.

I have one point that would need further clarification in the text. I think that at ll. 387-409 there is a conceptual misunderstanding when the authors want to demonstrate that the X-ray dose did not or did only minimally affect the embolism recovery capability. Here in this new version of the manusctipt the authors looked at rates of water potential recovery, to justify that the MicroCT treatment had only marginally affected the hydraulic recovery outcome. However, having a similar water potential recovery (and root water uptake) does not necessary imply that the MicroCT X-ray dose could not have affected the cellular processes involved in the active xylem embolism repair process. In this case, we are talking about RNA integrity disruption, with negative consequences in protein synthesis, and therefore altering the cascade of processes (e.g sugar transport) that are thought to be involved in the embolism repair. For this reason, also lines 434-437 in the "results" part should be modified accordingly.

Two minor mistakes:
L. 392: the sentence should be either "an altered pattern" or "altered patterns" without "an.
Line 578: please substitute "show" with "shows" as the subject is "Work"

Response to reviewer letter

Thank you for your feedback on our manuscript PP2024-RA-00313D entitled “A one-way ticket: Can wheat roots functionally refill emboli after xylem embolism?”

The input of both the reviewers and the managing editor have been inviable. We are glad that the extra experimental work in our previous submission met with the approval of the reviewers. We believe this has helped make this a more comprehensive piece of literature.

Thank you for your comments regarding the recent submission. We are pleased to make all changes suggested, and have attached a list of each modification made to the manuscript below. The corresponding changes can be found in the associated “revised manuscript text file with track changes” document.

Response to reviewer 1:

We are glad that you approve of the changes to the work made in our previous submission, and that you agree with the overall findings now. We agree with your comment that the degree of imposed stress is severe and needs acknowledgement. We have modified the abstract for increased specificity.

In address of this point, the following change has been made:

- Abstract: the summary statement now reads “Using MicroCT, and Optical and dye staining methods we investigate embolism repair in rehydrated intact roots of wheat (*Triticum aestivum* L.'Krichauff') exposed to a severe water deficit of -3.5 MPa, known to cause approximately 30 % total root network embolism in this species”.

Response to reviewer 2:

Thank you for your invaluable feedback, both in your previous comments and in your comments here. We are glad that our previous changes met with your approval, and we are also glad that the wording surrounding the subtraction method has been improved for clarity.

We appreciate your feedback regarding the wording and conceptual explanation of the X-ray exposure, and agree with your point that there is still likely to be a degree of disruption of metabolic processes. We believe there is still some utility to keeping the rate of recovery data in the supplement, but we have included an additional cautionary statement at the end of this passage given your comments. The additional statement now reads:

- Line 409: “However, these data cannot exclude the possibility that X-ray dose may have affected the cellular processes (RNA integrity and protein synthesis) as shown by Petruzzellis et al. (2018), potentially involved in the active xylem embolism repair process, so that the MicroCT data should still be treated with some caution as a standalone finding”. We hope that this meets with your approval.
- We have removed the statement in the results on lines 434-437 (now 436-438) given your comments. We have replaced this with the more cautious statement: “These

MicroCT data should be considered with a degree of caution in case repeat X-ray exposure may have impeded cellular processes associated with active water uptake in living root cortical cell layers".

Additionally, we have corrected the following points:

- L. 392: "an" has been removed so that this sentence now states "potentially resulting in altered patterns of root water".
- Line 580: "show" has been replaced with "shows" so that the sentence now states "Work on other angiosperms using non-invasive techniques also shows that embolism resistance…".

21-Jun-2024
Ms. Beatrice Linda Harrison Day
University of Tasmania
School of Natural Sciences
Hobart, TAS 7000
Australia

MSID: PP2024-RA-00313DR1
MS TITLE: A one-way ticket: Can wheat roots functionally refill xylem emboli following rehydration?

Dear Dr. Beatrice Harrison Day:

I am pleased to inform you that your revised manuscript "A one-way ticket: Can wheat roots functionally refill xylem emboli following rehydration?" has been accepted for publication in Plant Physiology. Your manuscript will be checked for consistency with journal formatting and image standards by one of our Science Editors, who will contact you shortly. They may have a number of queries and suggestions for you. Please keep in mind that they work to increase the online visibility of your article and its attraction for readers from the widest of backgrounds. You will have the opportunity to review any revisions, and I hope you will work constructively with them to get the most out of your research. You will also have the opportunity to review all changes when you receive the author proofs for your article.

Once validated for publication, your article will be sent to a Science Editor for a final review of your manuscript and source files. After your final files are uploaded, your article will appear in the next available issue and the online Preview version of your article will be posted after your signed Copyright and Checklist forms and final source files are received.

Please be sure to include the following in your manuscript file:
Harvard style citations
List of all author contributions and funding information at the beginning of manuscript after the titles and author list.
List all supplemental materials associated with the article, with titles, at the end of the ms, before the references and acknowledgments section.
Complete Figure Legends for all figures appearing in the article listed before the Literature Cited.

Thank you and congratulations on your Plant Physiology paper!

Sincerely,
Editor, Plant Physiology

=====
FIRST AUTHOR PROFILES
=====

We recognize the hard work that goes into being First Author of a Plant Physiology paper by publishing a "First Author Feature" for each First Author alongside your paper.

If the first author(s) would like to submit information for a feature on Facebook and Twitter, fill out the form by clicking on the link below:

Plant Physiology First Author Feature Form: https://forms.office.com/r/ZBCGZfhm8F
If you cannot access the form through the link above, please contact Rachel at rbelsky@aspb.org.

To upload an image of yourself to accompany your profile, please put your photo in the correct journal folder at the link below. You must label your image: Author First Name Last Name Article Title.

First Author Profile Image Dropbox: https://www.dropbox.com/scl/fo/xz029p7qjr2jml2rp2kmq/h?dl=0&rlkey=6fkxcaqmxx35ap152b2osy1fz

=====

IMPORTANT REMINDER: PEER REVIEW REPORTS
=====

If you opted to publish a peer review report along with your article during the original submission process, it will be prepared by the editorial staff and publicly posted with your manuscript, inside the zip file that contains any other supplemental material. As a reminder, the peer review report is a public record of all comments from editors and reviewers, as well as your prior responses, as you received them in the decision letters for each draft of your manuscript. If you agreed to publish this report and have changed your mind, or are not sure if you selected this option, please contact the editorial office as soon as possible before signing the license agreement from our publisher.

-- Reviewer comments:
